# The extinct shark, *Ptychodus* (Elasmobranchii, Ptychodontidae) in the Upper Cretaceous of central-western Russia—The road to easternmost peri-Tethyan seas

**DOI:** 10.1080/02724634.2022.2162909

**Published:** 2023-02-09

**Authors:** Manuel Amadori, Sergey V. Solonin, Alexey V. Vodorezov, Ryan Shell, Robert Niedźwiedzki, Jürgen Kriwet

**Affiliations:** 1 University of Vienna, Department of Palaeontology, UZAII, Geozentrum, Josef-Holaubek-Platz 2, Vienna, 1090, Austria; 2 Department of Geography, Ecology and Natural Management, Ryazan State University named for S. Yesenin, Ryazan, 390000, Russia; 3 Department of Vertebrate Paleontology, Cincinnati Museum Center, Cincinnati, 45203, U.S.A; 4 Institute of Geological Sciences, University of Wrocław, Wrocław, 50-204, Poland

## Abstract

Isolated teeth belonging to the genus *Ptychodus* Agassiz, 1834 (Chondrichthyes; Elasmobranchii) from the Upper Cretaceous of the Ryazan and Moscow Oblast regions (European Russia) are described and discussed in detail herein. The taxonomic composition of the *Ptychodus* assemblage from the Ryazan region is very diverse including the first records of the cuspidate species *P*. *altior* and *P*. *anonymus*, which thus is largely consistent with those from other contemporaneous European localities. *Ptychodus* ubiquitously inhabited epicontinental seas of Europe during most of the Cretaceous with the most diverse assemblages coming from southern England, northern Italy, Belgium, and European Russia. Additionally, the material documented here from the Cenomanian of Varavinsky ravine area (Moscow Oblast) represents the northernmost occurrence of *Ptychodus* hitherto reported from Europe. It is evident that the Late Cretaceous shallow seas of the Russian platform represented a crucial pathway for the dispersal of *Ptychodus* from the European peri-Tethys to the eastern margins of the Neo-Tethyan Ocean. The Albian–Campanian records of *Ptychodus* from Europe indicate that its dominance in the peri-Tethys persisted for most of its evolutionary history. A local temperature drop across most of the European shallow seas probably contributed to the narrowing of its geographic range in the peri-Tethyan seas towards the end of the Mesozoic Era. The fossil remains of *Ptychodus* documented herein are accordingly of utmost importance for better understanding the taxonomic composition of Russian fossil ichthyofaunas and also inform about the dispersal of *Ptychodus* towards western and eastern peri-Tethyan seas during the Late Cretaceous.

## Introduction

The Late Cretaceous represents a time of tremendous global changes in the history of Earth, such as paleogeographic modifications and climate fluctuations, with relevant implications in the course towards current conditions (e.g., Núñez-Useche et al., 2014; Stanley and Luczaj, 2015). In particular, the Cretaceous global and regional shifts in available aquatic habitats driven by these environmental and climatic changes probably affected the geographic distribution and the evolution of marine communities (e.g., Kriwet and Klug, 2008; Peterson and Lieberman, 2012; Sorenson et al., 2014; Poyato-Ariza and Martin-Abad, 2016). Additionally, Late Cretaceous elasmobranchs particularly experienced one of the greatest bursts of diversity in their evolutionary history, also developing various trophic adaptations and strategies (e.g., durophagous feeding; Walker and Brett, 2002; Summers et al., 2004; Underwood, 2006; Sorenson et al., 2014).

**Figure 1:**
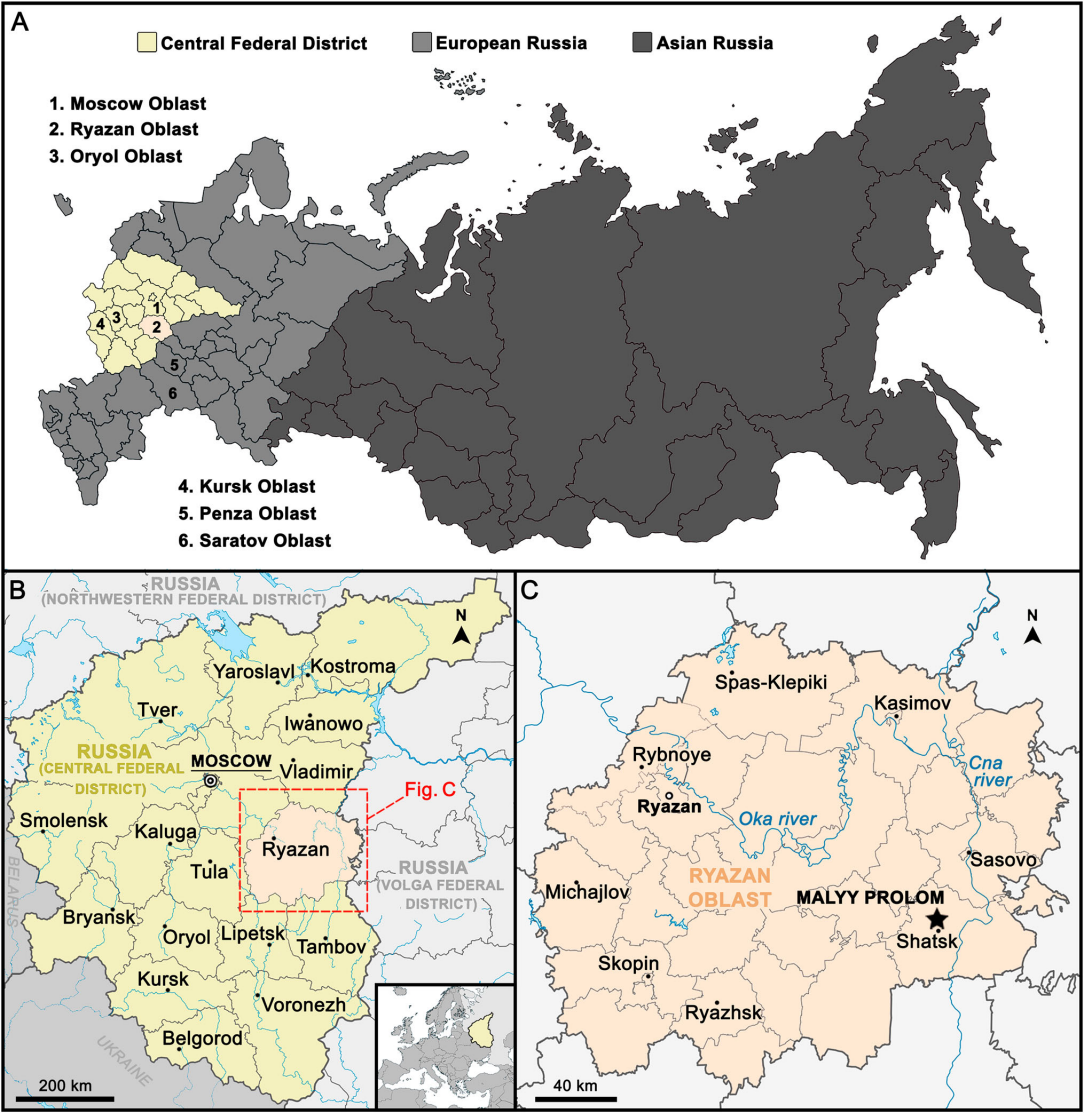
Locality maps showing approximate area of European Russia (**A**) with localities where *Ptychodus* has been hitherto reported (n.1-5), the Central Federal District (**B**), and the Ryazan Oblast (**C**; black star, Malyy Prolom quarry).

The northeastern peri-Tethys represented a crucial connection between the various peripheral areas of the Neo-Tethys Ocean with the Russian Platform during the Late Cretaceous that had a major role in shaping aquatic environments, and possibly the distribution of marine faunas, across these epicontinental sea areas (e.g., Baraboshkin et al., 2003; Solonin et al., 2021b; Zorina, 2022). Sharks from the Upper Cretaceous of central Russia have not yet been extensively studied and those studies available mainly are limited to Cenomanian deposits (Kiprijanoff, 1852, 1881; Glikman, 1980; Eremin, 1998; Olferev and Alekseev, 2005; Starodubtseva et al., 2008). Kiprijanoff (1852) reported for the first time elasmobranch faunas from the Cenomanian of Kursk Oblast (western European Russia). Later, elasmobranch teeth were described from the Upper Cretaceous of several other localities of European Russia (e.g., Moscow, Saratov, and Volga areas; Glikman, 1980; Eremin, 1998; Olferev and Alekseev, 2005; Starodubtseva et al., 2008). Recently, Solonin et al. (2020) provide a first preliminary assessment of an elasmobranch fauna, including *Ptychodus*, from the Ryazan Oblast area (European Russia; Vodorezov and Solonin, 2017; see also Fig. 1).

This extinct shark taxon of uncertain relationships currently is placed into its own family, Ptychodontidae. It is predominantly known from Cretaceous deposits of North America, Europe, Africa, and Asia with a fossil range from the Albian to the Campanian (e.g., Cappetta, 2012). The remains typically consist of isolated teeth and associated or articulated dental plates; there are also rare reports of mineralized cartilaginous elements mostly consisting of scattered vertebral centra and jaw rami (Woodward, 1887, 1912; Niedźwiedzki and Kalina, 2003; Everhart and Caggiano, 2004; Cappetta, 2012; Amadori et al., 2020a; Hamm, 2020a). In general, the heterodont, ‘pavementlike’ dentitions of *Ptychodus* are composed by variously ornamented molariform teeth (durophagous adaptations), which characterize this elasmobranch predator (Woodward, 1912; Cappetta, 2012; Shimada, 2012; Amadori et al., 2020a, 2020b, 2022). In particular, flat to cuspidate teeth probably allowed them to feed on a wide range of prey, including shell-covered cephalopods and other molluscs, and reach considerable sizes (Niedźwiedzki and Kalina, 2003; Cappetta, 2012; Shimada, 2012; Diedrich, 2013; Amadori et al., 2019b, 2020a, 2022; Hamm, 2020b). Although the taxonomic identification of *Ptychodus* species is traditionally based on peculiar, well-recognizable dental features, both higher-level and species-level systematics of this enigmatic elasmobranch group are still debated (Nicholls, 2010; Cappetta, 2012; Shimada, 2012; Amadori et al., 2019b, 2020a; Hamm, 2019).

In the present paper, we present the first detailed assessment of a very diverse and relatively abundant ptychodontid assemblage from the Upper Cretaceous of European Russia. In particular, isolated teeth of *Ptychodus* from the ?Cenomanian–Santonian of the Malyy Prolom area (Ryazan Oblast) are documented and discussed in detail herein, contributing to our knowledge of Upper Cretaceous elasmobranch faunas from European Russia. Moreover, unknown fragmentary teeth of *Ptychodus* from the Cenomanian of the Varavinsky ravine (Moscow Oblast) are described here for comparative purposes. The species richness of various Russian ptychodontid assemblages is also compared here with those of the main European elasmobranch faunas including *Ptychodus* that previously were documented. We therefore also present short reviews of European occurrences of *Ptychodus*. Additionally, we propose a hypothesis on possible dispersal patterns of *Ptychodus* towards the northeastern peri-Tethyan basin during the Late Cretaceous and tentatively correlate possible migration patterns with possible abiotic factors.

## Previous Records of *Ptychodus* European Russia

Cretaceous ptychodontids from European Russia have not been studied in adequate detail up to now. The first description of teeth of *Ptychodus* from central Russia dates back to the second half of the 19th century (Kiprijanoff, 1852, 1881; Nikitin, 1888; Sinzow, 1899). However, there are only very few reports on findings of ptychodontid remains from central Russia in the following decades. Kiprijanoff (1852) established the presence of *P*. *latissimus* Agassiz, 1835, and *P*. *polygyrus* Agassiz, 1835, from the Cretaceous sandy marls or sands in the Kursk Governorate (now Kursk Oblast), and *P*. *decurrens* Agassiz, 1838, *P*. *latissimus*, *P*. *mammillaris* Agassiz, 1835, from the same deposits in Oryol Governorate (now Oryol Oblast). Later, Kiprijanoff (1881) added information about new findings of *P*. *oweni* Dixon, 1850, and *P*. *decurrens* teeth from the same lithostratigraphic level in Oryol Oblast. All the above-mentioned specimens of *Ptychodus* were found directly below or directly above the so-called ‘Kursk samorod’ (Kiprijanoff, 1852) or ‘Siwerischen Osteolith’ (Kiprijanoff, 1881). Both terms are synonyms used to describe a layer of quartz-glauconite sands with numerous phosphorite pebbles. Based on their foraminiferous content, the layer is dated as early Cenomanian (e.g., Olferev and Alekseev, 2005).

Subsequently, Sinzow (1899) described a taxonomically diverse ptychodontid assemblage (*P*. *decurrens*, *P*. *mammillaris*, *P*. *polygyroides* Sinzow, 1899, *P*. *polygyrus*, and *P*. *rugosus* Dixon, 1850) from Cenomanian sandstones and sands with intercalated phosphorite horizons in the vicinity of the city of Saratov (now Volga Federal District). New research has led to the discovery of other outcrops with *Ptychodus* teeth in Saratov Oblast (Glickman, 1953; Biriukov, 2018) and added *P*. *latissimus* to Sinzow’s (1899) list of Cenomanian species (Glickman, 1953). Additionally, teeth of *P*. *concentricus* (?*P*. *marginalis*), *P*. *deccurrens* (sic), *P*. *latissimus*, *P*. *mammillaris*, and *P*. *multistriatus* (now *P*. *decurrens*) were found in ?upper Cenomanian deposits of the Saratov region (see Glickman, 1955). Biriukov (2018) mentioned the occurrence of *P*. *decurrens* teeth in the phosphorite horizon in middle Cenomanian sands in Penza Oblast. Some authors (Glickman, 1955; Mertinene, 1982; Biriukov, 2018) stated that *Ptychodus* species have a stratigraphic significance at least in the Cenomanian of the Volga River Basin.

*Ptychodus* teeth also occur in the northern part of Moscow Oblast where teeth of *P*. *polygyrus*, *P*. *mammillaris*, and *Ptychodus* sp. were found in quartz sands with phosphorite concretions in the Varavinsky ravine (near Sergiev Posad). Based on ammonites, the layer is dated as early–middle Cenomanian (Nikitin, 1888; Olferev and Alekseev, 2005). *Ptychodus* teeth are rare in all the above–mentioned fossil sites and coexist with much more abundant assemblages of teeth belonging to various other elasmobranch groups (compare Glickman, 1953; Olferev and Alekseev, 2005; Starodubtseva et al., 2008; Popov, 2016).

## Geological Setting

In general, the Upper Cretaceous stratigraphy of European Russia is quite problematic due to the regular erosion of sediments, which had gradually accumulated from the Cenomanian to the Santonian. Periodic regressions of the sea might have occurred, during which sediments were eroded and fossils (e.g., shark teeth) re-deposited at the base of the strata. The wide-spread marine transgression in the early–middle Cenomanian was replaced by a significant regression in the late Cenomanian–early Turonian. This exposed and destroyed most Cenomanian deposits, after which numerous transgressive-regressive cycles began during the Turonian–Santonian (Baraboshkin et al., 2003; Solonin et al., 2021b).

Biostratigraphy of Albian–Santonian deposits of the central part of European Russia (e.g., the Moscow syncline) is based mainly on radiolarians and ammonites for Albian and lower Cenomanian rocks and on inoceramids for the upper Cenomanian–Santonian interval (Sidorenko, 1971; Sahagian et al., 1996; Olferev and Alekseev, 2005; Kuzmin et al., 2015).

### Ryazan Oblast (Malyy Prolom)

**Figure 2:**
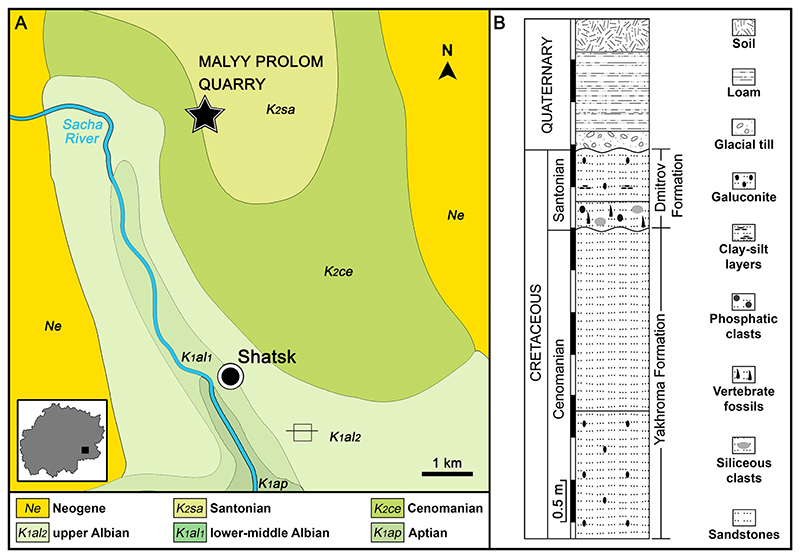
Mesozoic sedimentary strata (**A**; black star, Malyy Prolom quarry) and the stratigraphic column (**B**) depicting the Yakhroma and Dmitrov Formations sedimentary succession and fossil bearing horizon within the Malyy Prolom quarry exposure (modified after Solonin et al., 2021b).

The teeth of *Ptychodus* documented herein have been collected from an active sand quarry near the village of Malyy Prolom, located about 5.0 km northwest of Shatsk in the south-eastern part of Ryazan Oblast area (see Figs. 1, 2). The area of Ryazan Oblast geologically is located in the southeastern part (Chuchkovo depression) of the Moscow Syneclise in the central part of the Russian Platform (East European Platform; Kuzmin et al., 2015). Large stratigraphic gaps including Turonian–Coniacian and Campanian–Maastrichtian stages characterize the Upper Cretaceous stratigraphy of the Ryazan region (Sahagian et al., 1996; Fadeeva and Iosifova, 1998).

In the southeastern part of the Ryazan Oblast area (Chuchkovo depression with Malyy Prolom quarry) only four Upper Cretaceous formations are differentiated in the geological section: (1) the Yahroma Fm. (lower Cenomanian), (2) the Lyaminsk Fm. (upper Cenomanian), (3) the Zagorsk Fm. (?lower Santonian), and (4) the Dmitrov Fm. (upper Santonian; see Drutskoi and Fadeeva, 2001; Olferev and Alekseev, 2005; Kuzmin et al., 2015). Both Cenomanian formations and the Dmitrov Fm. were deposited in marine shoreface conditions, while the Zagorsk Fm. were formed in offshore–deep marine conditions (Sahagian et al., 1996).

In the Malyy Prolom area, directly below Quaternary sediments, upper Santonian quartz sands and sandstones (Dmitrov Fm.) occur, which are underlain by lower Cenomanian quartz sands of the Yakhroma Fm. and upper Albian clays and silts of the Paramanovsk Fm., respectively (Drutskoi and Fadeeva, 2001; Kuzmin et al., 2015; see also Fig. 2). Deposits of different geological stages are separated by distinct erosional unconformities (Sidorenko, 1971; Nikitin et al., 1984; Kuzmin et al., 2015).

The Malyy Prolom quarry exposes 4.5 m fine-grained Cretaceous quartz sands or quartz-glauconite sands covered by 1.6 m of Quaternary sediments (Fig. 2). Cretaceous rocks do not contain body fossils with the exception of a layer of coarsegrained sand in the upper part of this unit (0.1–0.6 m). Its paleontological content mainly includes vertebrate remains consisting of numerous elasmobranch and actinopterygian teeth, rare vertebrae as well as extremely rare teeth of marine reptiles (plesiosaurians, ichthyosaurians, mosasaurians) and pterosaurs (see also Solonin et al., 2021a, 2021b). In the same layer, rare fragmentary remains of the bivalves (e.g., *Neithea* and pectenids), sponges, bryozoans, calcareous algae, and fragments of ichnofossil burrows were documented (see also Solonin et al., 2021a, 2021b). This fossiliferous layer also differs from underlying and overlying sand layers in larger grain diameters and the presence of phosphatic pebbles of sandstones and large siliceous sandstone concretions. This fossiliferous layer is the only Cretaceous bed, which lies on a strongly eroded surface (Krivtsov et al., 2018).

Unfortunately, the Cretaceous sands of the Malyy Prolom quarry have not yielded any stratigraphically important taxa up to now. However, analyses of the available geological maps of the region (Fadeeva and Iosifova, 1998; Kuzmin et al., 2015) and geological profiles of nearby boreholes from Karnauhovo (3 km east of the studied quarry) prove that the uppermost part of the Cretaceous sands (including the fossiliferous layer) of the Malyy Prolom quarry, which is separated from underlying beds by an erosional surface, is certainly late Santonian (Dmitrov Fm.) in age, while the underlying sand layers are of early Cenomanian (Yahroma Fm.) age (see Fig. 2).

The low degree of preservation exhibited by the fossils from the Upper Cretaceous of Ryazan Oblast (European Russia) indicates post-mortem re-deposition for most of these specimens (see the ‘Taphonomic remarks’ section, below). However, re-deposition events could also have involved better-preserved teeth, such as those described here for *Ptychodus*. Although previous preliminary analyses indicate the presence of Santonian nannofossils embedded within the sandstone from which the examined teeth come, the age of the specimens cannot be conclusively established (Olferev and Alekseev, 2005). The teeth of *Ptychodus* from the Ryazan Oblast are therefore considered here to be ?Cenomanian–Santonian in age.

### Moscow Oblast (Varavinsky Ravine)

Varavinsky Ravine is located about 1 km northeast of the village of Varavino in the north part of Moscow Oblast. Here, the geologic section of Upper Cretaceous deposits is represented by Cenomanian sands of the Yakhroma Fm. (Lower Cenomanian) and the Lyaminsk Fm. (Upper Cenomanian) and by the sands, silts, and clays of the Zagorsk Fm. (Coniacian–lower Santonian). Shark teeth of *Synechodus dispar, Eostriatolamia subulata, Scapanorhynchus raphiodon*, *Pseudoisurus tomosus*, *P*. *macrorhizus*, and *Ptychodus* sp. have been recovered in the deposits of the Yakhroma Fm. (Olferev and Alekseev, 2005).

## Materials and Methods

**Institutional Abbreviations**—**RSU DGE**, Ryazan State University (Department of Geography, Ecology and Natural Management, Ryazan Oblast), Russia; **SS106**, Sasovo Secondary School N. 106, Russia; **KP NVF**, State Darwin Museum in Moscow, Russia.

### Materials

All the material documented here was collected from the Malyy Prolom sand quarry in the Ryazan Oblast (western European Russia). This collection consists of 69 specimens in total, and comprise complete, broken, and fragmentary teeth (catalog numbers: RSU DGE 2018 RO MP-41–RSU DGE 2018 RO MP-47, RSU DGE 2019 RO MP-43, RSU DGE 2020 RO MP-3, RSU DGE 2020 RO MP-6–RSU DGE 2020 RO MP-14, RSU DGE 2020 RO MP-16–RSU DGE 2020 RO MP-29, RSU DGE 2020 RO MP-38, RSU DGE 2020 RO MP-39, RSU DGE 2021 RO MP-1–RSU DGE 2021 RO MP-30, SS106-1–SS106-4, and SS106-7). All specimens are housed in the collections of the Ryazan State University (RSU) and the Sasovo Secondary School N. 106 (SS106).

In addition, six teeth (catalog numbers: KP NVF 19956/16, KP NVF 19956/214–KP NVF 19956/218) of *Ptychodus* from the Cenomanian of the Varavinsky ravine locality (Moscow Oblast, western European Russia) and housed in the collection of the State Darwin Museum in Moscow were included as comparative material (see Tables S1 and S2 in Supplemental Data, for more details on all examined material).

### Methods

The material described herein was obtained by bulk sampling and subsequent screen washing of fossiliferous sediments from the Malyy Prolom sand quarry with the use of sieves of different mesh sizes (0.5–5 mm). Some of the specimens were collected using a BM-51-2 binocular stereo microscope (magnification 8.75×) from concentrates of the sieved samples, while others were surface-collected. All specimens were cleaned with water.

Specimens were photographed using a Nikon D7000 DSLR camera with an 18–55 mm Nikon lens and a Canon EOS 650 DSLR camera with an 18–55 mm Canon lens. Illustrative drawings, images of the specimens and graphics were prepared using the software packages Past (v. 3.25), Microsoft Excel 2013 (v. 15.0.5415.1000), GIMP (v. 2.8.16), and Adobe Photoshop CC 2017 (v. 2017.04.25.r.252).

In order to ensure a rigorous comparison, five Russian localities have been selected (Moscow Oblast, Ryazan Oblast, Oryol Oblast, Kursk Oblast, and Saratov Oblast) and the remaining European occurrences of *Ptychodus* were subdivided into 19 geographic areas (southern Spain, southern England, northern France, western France, southeastern France, Belgium, western Germany, eastern Germany, northern Czech Republic, northern Switzerland, eastern Austria, northern Italy, central Italy, southern Italy, southern Sweden, Denmark, southern Poland, central Lithuania, and central Romania). Dubious occurrences with non-described and/or non-figured specimens have been discarded for the species richness comparison. The anatomical and odontological terminologies mostly follow Cappetta (2012), Shimada (2012), and Amadori et al. (2019b, 2020a). Synonymy lists follow the standards proposed by Matthews (1973), Bengtson (1988), and Sigovini et al. (2016), but are not intended to be complete; only important references are listed.

## Systematic Paleontology

Class CHONDRICHTHYES Huxley, 1880

Subclass ELASMOBRANCHII Bonaparte, 1838

Order PTYCHODONTIFORMES Hamm, 2019

Family †PTYCHODONTIDAE Jaekel, 1898

Genus †*PTYCHODUS* Agassiz, 1834

**Type Species**—*Ptychodus latissimus* Agassiz, 1835 (nomen protectum; see Giusberti et al., 2018).

**Diagnosis**—See Woodward (1912).

### †Ptychodus Altior Agassiz, 1835 (Fig. 3)

(Selected synonyms)

p.“Teeth allied to *Diodon*”: Mantell, 1822:231, pl. 32, figs.17, 21, 27 (non figs. 18–10, 23–25, 29).

†*Ptychodus altior* Ag.: Agassiz, 1835:54 (Feuilletton additionnel).

v*Ptychodus altior* Agassiz 1835: Amadori et al., 2019b:4, figs. 1, 3–11, 13, 14 (cum syn.).

*Ptychodus altior* Agassiz 1835: Hamm, 2020a:38, fig. 61 (non syn.).

v*Ptychodus altior*: Solonin et al., 2020:fig. 5.1a, b.

#### Diagnosis

See Amadori et al. (2019b:5).

#### Species Stratigraphic Range

*Ptychodus altior* is well known from middle Turonian to Coniacian (see Hamm, 2020a:fig. 100).

#### Referred Material

Seven isolated teeth, mostly without roots (RSU DGE 2018 RO MP-47, RSU DGE 2020 RO MP-8, RSU DGE 2020 RO MP-9, RSU DGE 2020 RO MP-17, RSU DGE 2020 RO MP-25, RSU DGE 2020 RO MP-39, and RSU DGE 2021 RO MP-1) and two tooth fragments (RSU DGE 2020 RO MP-27 and RSU DGE 2021 RO MP-16) belonging to the fossil collections of the Ryazan State University.

#### Description

**Figure 3:**
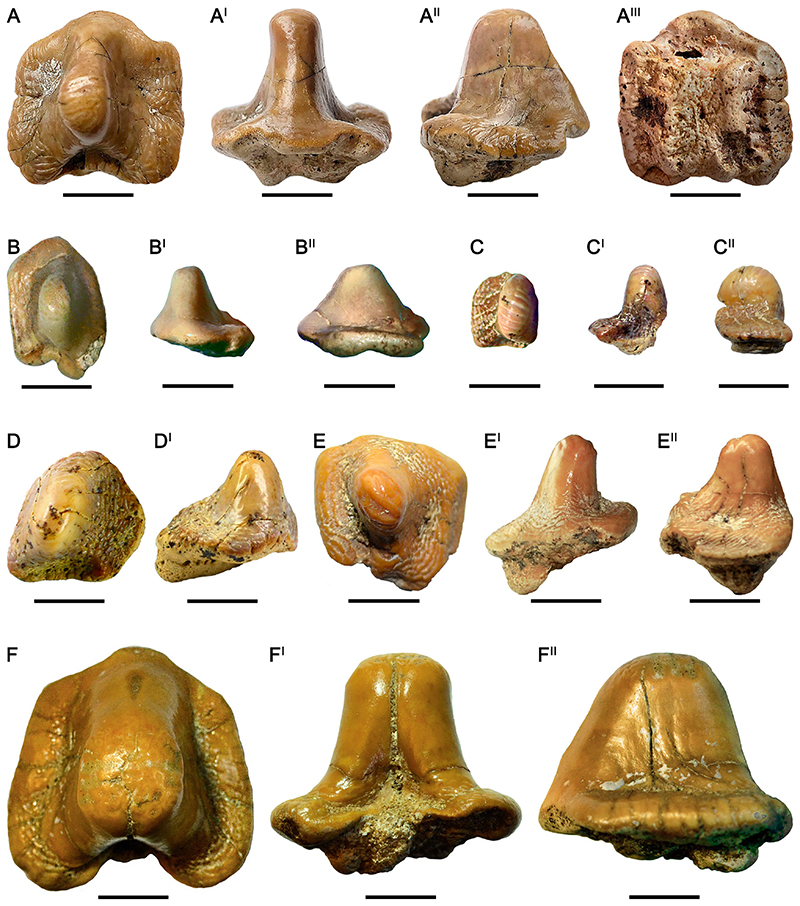
Teeth of *Ptychodus altior* Agassiz, 1835, from the Upper Cretaceous of Ryazan Oblast (western Russia) in occlusal (**A**, **B**, **C**, **D**, **E**, **F**), anterior (**A^I^**, **B^I^**, **D^I^**, **E^1^**) posterior (**C^I^**, **F^I^**), lateral (**A^II^**, **B^II^**, **C, E^II^**, **F^II^**), and inferior (**A^III^**) views. **A-A^III^**, RSU DGE 2018 RO MP-47; **B-B^II^**, RSU DGE 2020 RO MP-5; **C-C^II^**, RSU DGE 2020 RO MP-8; **D, D^I^**, RSU DGE 2020 RO MP-9; **E-E^II^**, RSU DGE 2020 RO MP-17; **F-F^II^**, RSU DGE 2021 RO MP-1. Scale bars equal 5 mm.

The isolated teeth RSU DGE 2018 RO MP-47 (Fig. 3A–A^III^), RSU DGE 2020 RO MP-8 (Fig. 3C–C^II^), RSU DGE 2020 RO MP-9 (Fig. 3D–D^I^), RSU DGE 2020 RO MP-17 (Fig. 3E–E^II^), RSU DGE 2020 RO MP-25, RSU DGE 2020 RO MP-39 (Fig. 3B–B^II^), and RSU DGE 2021 RO MP-1 (Fig. 3F–F^II^) share general crown features with each other. RSU DGE 2018 RO MP-47 has an almost quadrangular occlusal outline (see Fig. 3A). The left side of the dental crown is barely subdivided in two rounded ‘marginal lobes’ (posterior and anterior) by a slight, shallow sulcus, whereas the right margin is almost straight. This isolated tooth exhibits a unique narrow, occlusal cusp characterized by smooth, inclined sides and a rounded apex with slight ornamentation (see Fig. 3A^I^). A deep posterior sulcus characterizes the posterior side and a well-developed anterior protuberance elongates the anterior margin of the tooth crown. The tooth apex is almost completely abraded, whereas the marginal ornamentation exhibits an almost concentric pattern around the cusp (see Fig. 3A). In lateral view (Fig. 3A^II^), the dental cusp is well-developed with an anterior outline markedly inclined and a posterior side almost perpendicular to the crown base. A small portion of tooth root still is preserved. In occlusal view (Fig. 3B), RSU DGE 2020 RO MP-39 shows an irregular and asymmetrical crown outline with a left marginal area almost straight and a curved, convex tooth edge on the right one; the latter is slightly damaged posteriorly. A deep and narrow posterior sulcus characterizes the posterior side. The marginal ornamentations consisting of fine roughness and granules are unmarked and do not show a diagnostic pattern. Slight abrasions are apparent on the tooth apex (Fig. 3B). The dental root is missing. In RSU DGE 2020 RO MP-8, both anterior and right marginal areas are missing (see Fig. 3C–C^II^). The apical ornamentation consists of at least seven slight parallel ridges (Fig. 3C). The tooth wear is present on most of the ridges. In lateral view (Fig. 3C^II^), several foramina are recognizable under the base of the dental crown. RSU DGE 2020 RO MP-9 (Fig. 3D–D^II^) and RSU DGE 2020 RO MP-25 both have a markedly asymmetrical outline in occlusal view. The marginal area is poorly developed on the left side with a rounded outline, whereas the right side is enlarged and curved posteriorly. The dental wear has completely abraded the apical ridges on the tooth cusps. Spotted, dark traces are easily recognizable and spread across the entire dental crown of RSU DGE 2020 RO MP-9 (Fig. 3D–D^II^). RSU DGE 2020 RO MP-17 (Fig. 3E–E^II^) has a well-developed cusp with apical ornamentation with at least three apical ridges. In RSU DGE 2020 RO MP-27, only the uppermost portion of the cusp is still preserved. The cusp sides are clearly smooth, whereas the apex exhibits 4–5 parallel, slight ridges. RSU DGE 2021 RO MP-1 (Fig. 3F–F^II^) is almost identical to RSU DGE 2018 RO MP-47 (Fig. 3A–A^III^); nevertheless, the ratio between apex and base of the cusp is higher in RSU DGE 2021 RO MP-1 (about 0.6) than in RSU DGE 2018 RO MP-47 (about 0.4). The rounded cusp apex of RSU DGE 2021 RO MP-1 is proportionally wider than that of RSU DGE 2018 RO MP-47. Moreover, only RSU DGE 2021 RO MP-1 has a symmetrical occlusal outline with the anterior margin slightly narrower than the posterior one (see Fig. 3F). In RSU DGE 2021 RO MP-16 the cusp apex and root are missing; the basal portion of the cusp is completely smooth. Marked anterior protuberance and posterior sulcus characterize its symmetrical outline. Concentric wrinkles cover the marginal areas and both lateral tooth edges are curved backwards.

#### Remarks

The tooth crown of the isolated specimens identified as belonging to this species is generally well preserved with few exceptions (e.g., RSU DGE 2020 RO MP-27). Likely, the teeth all belong to lateral rows of the dentition with the exception of RSU DGE 2021 RO MP-1. The latter is probably a symphyseal tooth due to its symmetrical crown outline. RSU DGE 2021 RO MP-1 (Fig. 3F–F^II^) exhibits a rounded, broad dental cusp similar to those characterizing *Ptychodus rugosus* (e.g., Hamm 2020:figs. 72–75; Amadori et al. 2019b:fig. 15A–A^II^). Nevertheless, RSU DGE 2021 RO MP-1 exhibits completely smooth cusp sides, which is a species-specific feature of *P*. *altior*. The symmetric outline of RSU DGE 2021 RO MP-16 indicates its original placement along the medial tooth rows; in general, its occlusal shape resembles that depicted in fig. 11, pl. 25b by Agassiz (1838). The features characterizing the upper and the lower dental plates of *P*. *altior* have not been hitherto defined due to the lack of articulated dentitions. The attribution to the maxillary or mandibular tooth plate thus remains doubtful for the teeth documented herein. The occlusal wear on the cusp apex of some teeth confirms their involvement in prey processing (see ‘functional teeth’ sensu Shimada, 2012; see also Amadori et al., 2019b, 2020a).

### †Ptychodus Anonymus Williston, 1900a (Fig. 4)

(Selected synonyms)

p.*Ptychodus anonymus*: Williston, 1900a:32, pl. 11, figs. 5, 6, 16–18, 24 (non figs. 7, 8, 21–22).

p.*Ptychodus anonymus*: Williston 1900b:241, pl. 29, figs. 5, 6, 16–18, 24 (non figs. 7, 8, 21–22).

*Ptychodus mammillaris – anonymus*: Herman, 1977:61, text fig. on p. 61 (right), pl. 2, fig. 5a–e

*Ptychodus anonymus* Williston 1900a: Hamm, 2020a:14, figs. 21–26.

#### Diagnosis

See Hamm (2020a:14).

#### Species Stratigraphic Range

The temporal distribution currently known for *Ptychodus anonymus* is early Cenomanianlate Turonian (see Hamm, 2020a:fig. 100).

#### Referred Material

Twenty-one isolated teeth (RSU DGE 2018 RO MP-42, RSU DGE 2018 RO MP-44, RSU DGE 2018 RO MP-46, RSU DGE 2020 RO MP-3, RSU DGE 2020 RO MP-7, RSU DGE 2020 RO MP-13, RSU DGE 2020 RO MP-14, RSU DGE 2020 RO MP-18–RSU DGE 2020 RO MP-20, RSU DGE 2020 RO MP-29, RSU DGE 2021 RO MP-3, RSU DGE 2021 RO MP-7; RSU DGE 2021 RO MP-8, RSU DGE 2021 RO MP-9, RSU DGE 2021 RO MP-12, RSU DGE 2021 RO MP-21, RSU DGE 2021 RO MP-23–RSU DGE 2021 RO MP-25, and KP NVF 19956/214) belonging to the fossil collections of the Ryazan State University and of State Darwin Museum in Moscow.

#### Description

**Figure 4:**
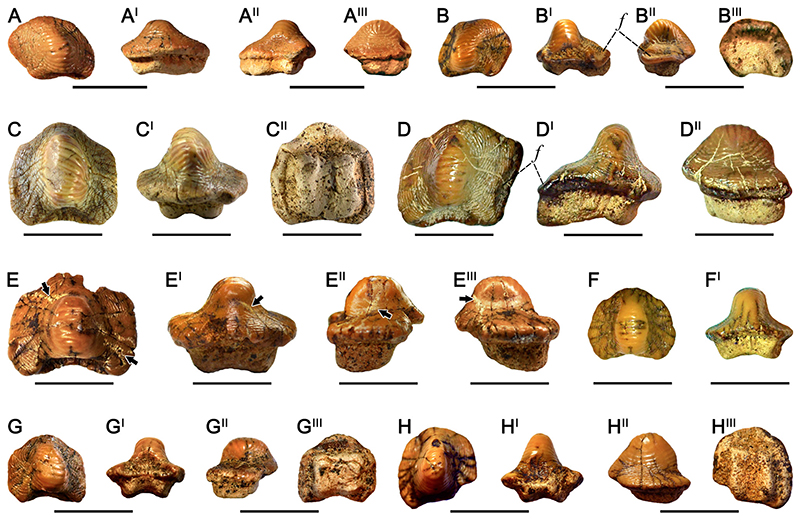
Teeth of *Ptychodus anonymus* Williston, 1900a, from the Upper Cretaceous of Ryazan Oblast (western Russia) in occlusal (**A**, **B**, **C**, **D**, **E**, **F**, **G**, **H**), anterior (**A^I^**, **B^I^**, **C^I^**, **D^I^**, **E^I^**, F*), posterior (**A^II^**, **G^I^**, **H^I^**), lateral (**A, B^II^**, **D^II^**, **E^II^**, **E**^III^, **G^II^**, **H^II^**), and inferior (**B**^III^, **C, G^III^**, **H^III^**) views. **A-A^III^**, RSU DGE 2018 RO MP-42; **B-B^III^**, RSU DGE 2020 RO MP-20; **C-C^II^**, RSU DGE 2020 RO MP-3; **D-D^II^**, RSU DGE 2021 RO MP-21; **E-E^III^**, RSU DGE 2020 RO MP-14; **F, F^I^**, RSU DGE 2021 RO MP-25; **G-G^III^**, RSU DGE 2021 RO MP-8; **H-H^III^**, RSU DGE 2021 RO MP-9. Scale bars equal 10 mm.

RSU DGE 2018 RO MP-42 (Fig. 4A–A^III^) exhibits an irregular, asymmetrical outline with the anterior and the right edges merged to each other, forming a single curved margin. In occlusal view (Fig. 4A), the anterior protuberance is rounded and the posterior sulcus is deep. The left margin of the crown is slightly concave. A rounded cusp crossed by six transversal, thin ridges characterizes the occlusal surface of the crown; concentric, thin wrinkles cover the entire marginal area. In posterior view (Fig. 4A^II^), the cusp is narrow and the crown base is flat; the root is bilobate with a wide, shallow antero-posterior sulcus (see also Fig. 4A^I^, A^II^). RSU DGE 2018 RO MP-44 and RSU DGE 2021 RO MP-7 have general features similar to RSU DGE 2018 RO MP-42 (Fig. 4A–A^III^). The left tooth edge in RSU DGE 2018 RO MP-44 is more straight and depressed; its cusps shows 6–7 thin ridges characterized by curved ends, which gradually merge with thin and concentric wrinkles covering the marginal area. The root of RSU DGE 2018 RO MP-44 is missing. The center of the occlusal surfaces of RSU DGE 2021 RO MP-7 is slightly abraded on the left. In anterior and posterior views, the crown is raised and pointed centrally but lower than in RSU DGE 2018 RO MP-42 (Fig. 4A^I^, A^II^). The tooth root of RSU DGE 2021 RO MP-7 is bilobate and characterized by a marked and wide antero-posterior sulcus. In lateral view, the anterior crown outline is inclined downwards, whereas the root is tilted posteriorly. RSU DGE 2018 RO MP-46, RSU DGE 2020 RO MP-7, RSU DGE 2020 RO MP-19, RSU DGE 2020 RO MP-20 (Fig. 4B–B^III^), RSU DGE 2020 RO MP-29, and RSU DGE 2021 RO MP-21 (Fig. 4D–D^II^) all show an asymmetrical crown with the cusp moved on the left side (in posterior view), a rounded anterior protuberance and a left dental edge curved backwards; the marginal area is well-developed and entirely covered by thin, concentric wrinkles. In RSU DGE 2018 RO MP-46 the anterior protuberance is pointed and the right side is straight. The dental root of RSU DGE 2018 RO MP-46 is poorly preserved. In occlusal view, RSU DGE 2020 RO MP-20 (Fig. 4B) and RSU DGE 2021 RO MP-21 (Fig. 4D) have a polygonal outline with the right tooth edges inclined posteriorly; the posterior sulcus is deep. Six to nine thin, transversal ridges cross the dental cusp of both teeth (see Fig. 4B, D). Their cusps are rounded and moved laterally; the lateral marginal areas are tilted downwards and the right tooth edges are depressed (see ‘*f*’ in Fig. 4B–B^II^, D–D^II^). The root is bilobate with a marked antero-posterior sulcus and the right lobe more developed than the other. In lateral view, the cusp of RSU DGE 2020 RO MP-20 (Fig. 4B^II^) and RSU DGE 2021 RO MP-21 (Fig. 4D^II^) shows an anterior outline tilted downwards. The root of RSU DGE 2020 RO MP-20 follows the crown outline in inferior view (Fig. 4B^III^). Slight traces of wear characterized the top of the cusp in RSU DGE 2020 RO MP-20 (Fig. 4B) and RSU DGE 2021 RO MP-21 (Fig. 4D). Although the right sides of RSU DGE 2020 RO MP-7, RSU DGE 2020 RO MP-19, and RSU DGE 2020 RO MP-29 are broken, they are similar to RSU DGE 2020 RO MP-20 (Fig. 4B–B^III^). RSU DGE 2020 ROMP-7 has a left marginal area well developed and transversally elongated. The root is missing in RSU DGE 2020 RO MP-7 and RSU DGE 2020 RO MP-19 and poorly preserved in RSU DGE 2020 RO MP-29. Both the lateral surfaces of the cusp apex of RSU DGE 2020 RO MP-7 exhibit slight traces of wear. The tooth wear in RSU DGE 2020 RO MP-19 involves the entire cusp apex, whereas it is limited to the anterior surface of the cusp apex in RSU DGE 2020 RO MP-29. RSU DGE 2020 RO MP-3 (Fig. 4C–C^II^) has an almost quadrangular and slightly asymmetrical crown with a left side more curved than the right one. Both the posterior sulcus and anterior protuberance are heavily marked. In occlusal view (Fig. 4C), 9–10 thin ridges cover the occlusal cusp, curving anteriorly on the side and merging with an almost concentric fine granulation. Most of the ridges on the cusp apex are abraded. In anterior view (Fig. 4C^I^), the cusp of RSU DGE 2020 RO MP-3 is well developed and blunt, whereas the bilobate root is characterized by a shallow antero-posterior sulcus. Both the lateral sides of the root are inclined inwards. In inferior view (Fig. 4C^II^), the tooth crown overhangs the rectangular root on all sides. RSU DGE 2020 RO MP-13, RSU DGE 2020 RO MP-18, RSU DGE 2021 RO MP-3, and RSU DGE 2021 RO MP-8 (Fig. 4G–G^III^) share general features with RSU DGE 2020 RO MP-3 (Fig. 4C–C^II^). Most of the right side of the RSU DGE 2020 RO MP-13 is missing and its outline seems quite symmetrical, also exhibiting a wide posterior sulcus. The cusp is rounded and crossed by around 10 thin ridges with lateral end curved anteriorly and merging to each other on the right side (‘loops’). The marginal area is covered by thin, concentric wrinkles. The crowns of RSU DGE 2020 RO MP-18 and RSU DGE 2021 RO MP-3 show approximately 11 occlusal ridges and all lateral edges rounded. The cusp of RSU DGE 2020 RO MP-18 is more developed and higher than that of RSU DGE 2020 RO MP-3 (Fig. 4C–C^II^). The root in RSU DGE 2020 RO MP-18 lacks the right lobe (in posterior view), whereas it is totally missing in RSU DGE 2021 RO MP-3. Six to seven thin ridges transversally cross the cusp of RSU DGE 2021 RO MP-8 (Fig. 4G). In occlusal view, RSU DGE 2021 RO MP-9 (Fig. 4H–H^III^) and RSU DGE 2021 RO MP-12 have a quadrangular outline with rounded lateral edges and deep posterior sulcus. Six to seven thin ridges transversally cross the cusp of RSU DGE 2021 RO MP-9 (see Fig. 4H). RSU DGE 2021 RO MP-12 shows approximately eight occlusal ridges. In posterior view, RSU DGE 2021 RO MP-9 (Fig. 4H^I^) has a high and narrow cusp; the cusp is lower in RSU DGE 2021 RO MP-12. The root of RSU DGE 2021 RO MP-9 is bilobate with a shallow antero-posterior sulcus and lateral sides inclined inwards (see Fig. 4H^I^), whereas it is missing in RSU DGE 2021 RO MP-12. In lateral view, both RSU DGE 2021 RO MP-9 (Fig. 4H^II^) and RSU DGE 2021 RO MP-12 have a cusp with the anterior side inclined downwards and the posterior outline almost perpendicular to the crown base; the root exhibits an anterior side tilted backward (see Fig. 4H^II^). RSU DGE 2020 RO MP-14 (Fig. 4E–E^III^), RSU DGE 2021 RO MP-23, RSU DGE 2021 RO MP-24, and RSU DGE 2021 RO MP-25 (Fig. 4F, F^I^) show a symmetrical outline with a deep posterior sulcus. The anterior protuberance of RSU DGE 2020 RO MP-14 (Fig. 4E) is partially broken, but well-developed. In RSU DGE 2020 RO MP-14 (Fig. 4E–E^III^), RSU DGE 2021 RO MP-24, and RSU DGE 2021 RO MP-25 (Fig. 4F, F^I^), around seven thin ridges cross the central cusp; concentric wrinkles cover the marginal area. In anterior view (Fig. 4E^I^), the cusp of RSU DGE 2020 ROMP-14 is rounded and relatively low, whereas the root is bilobate with a shallow antero-posterior sulcus. The right crown edge of RSU DGE 2021 RO MP-24 is broken. RSU DGE 2021 RO MP-25 (Fig. 4F, F^I^) displays a higher, rounded cusp. In anterior view (Fig. 4F^I^), the cusp is rounded and root is bilobate. On the occlusal surface of RSU DGE 2020 RO MP-14, whitish irregular traces are recognizable (see black arrows in Fig. 4E–E^III^). Moreover, marked abrasions characterize the entire cusp of both RSU DGE 2020 RO MP-14 (Fig. 4E–E^III^) and RSU DGE 2021 RO MP-25 (Fig. 4F, F^I^) with occlusal ridges deeply worn and barely observable. The crown apex of RSU DGE 2021 RO MP-23 is totally abraded. KP NVF 19956/214 is very similar to RSU DGE 2021 RO MP-9 (Fig. 4H–H^III^) a crown with a central, rounded cusp and two lateral lobes; the right tooth edge is broken. The cusp is entirely crossed by 9–10 thin ridges, whereas the marginal area shows a concentric ornamentation. The cusp apex is slightly abraded.

#### Remarks

Articulated dentitions of *Ptychodus anonymus* Williston, 1900a, are not presently known. Nevertheless, dental mesiodistal patterns are clearly recognizable in the associated tooth set FHSM-VP 19170 from the Upper Cretaceous Jetmore Chalk of Kansas (U.S.A.; see Hamm, 2019). The American tooth set exhibits symmetrical, symphyseal teeth with high cusps and lateral teeth with rounded and distally moved cusps (see also Hamm, 2020a:figs. 23, 24). The symmetrical outline, together with the central position of the dental cusp on the crown, would indicate the inclusion of RSU DGE 2020 RO MP-14 (Fig. 4E–E^III^), RSU DGE 2021 RO MP-23, RSU DGE 2021 RO MP-24, and RSU DGE 2021 RO MP-25 (Fig. 4F, F^I^) within the upper symphyseal tooth row. However, the low height of the cusp of RSU DGE 2020 RO MP-14 leaves doubts on the central position within the upper dental plate (see Fig. 4E^I^–E^III^). The cusp of RSU DGE 2021 RO MP-23, instead, is low and blunt due to marked wear. No other symmetrical teeth have been identified among the specimens of *P*. *anonymus* Williston, 1900a, here documented. All other teeth indeed show distally moved cusps, depressed areas on the mesial tooth edges, and distal marginal sides curved backwards. Based on the low degree of asymmetry exhibited by RSU DGE 2020 RO MP-3 (Fig. 4C–C^II^), RSU DGE 2020 RO MP-18, RSU DGE 2021 RO MP-3, RSU DGE 2021 RO MP-8 (Fig. 4G–G^III^), RSU DGE 2021 RO MP-9 (Fig. 4H–H^III^), RSU DGE 2021 RO MP-12, and KP NVF 19956/214, they likely were antero-lateral teeth and were probably arranged close to the symphyseal row. In particular, RSU DGE 2020 RO MP-18, RSU DGE 2021 RO MP-3, RSU DGE 2021 RO MP-8, and RSU DGE 2021 RO MP-12 would have been originally arranged within the right hemiarch, whereas RSU DGE 2020 RO MP-3 and RSU DGE 2021 RO MP-9 belong to the left hemiarch. RSU DGE 2018 RO MP-42 (Fig. 4A–A^III^), RSU DGE 2018 RO MP-44, and RSU DGE 2021 RO MP-7 were originally placed more distally within the right portion of the dentition. RSU DGE 2018 RO MP-46, RSU DGE 2020 RO MP-7, RSU DGE 2020 RO MP-19, RSU DGE 2020 RO MP-20 (Fig. 4B–B^III^), and RSU DGE 2020 RO MP-29 were arranged in distal tooth rows within the left hemiarch. Slight traces of wear have been observed in most of the teeth here documented. The low degree of tooth abrasion mainly limited on the dental cusp does not indicate an intense usage of the raised occlusal area for prolonged food processing and/or a fine shell grinding.

### † ptychodus decurrens Agassiz, 1838 (Fig. 5)

**Figure 5:**
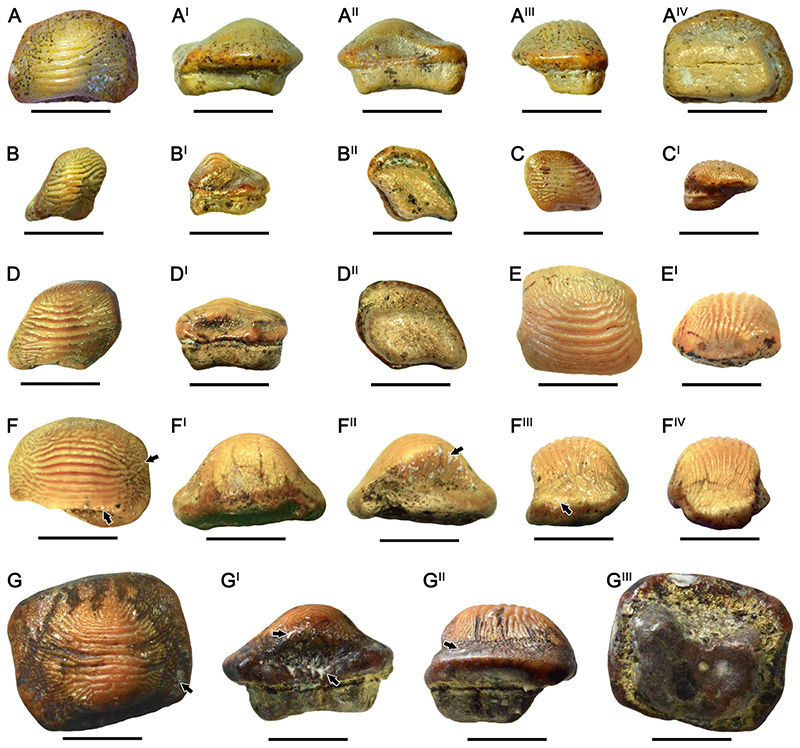
Teeth of *Ptychodus decurrens* Agassiz, 1837, from the Upper Cretaceous of Ryazan Oblast (western Russia) in occlusal (**A**, **B**, **C**, **D**, **E**, **F**, **G**), anterior (**A^I^**, **F^I^**), posterior (**A^II^**, **B^I^**, **D^I^**, **F, G^I^**), lateral (**A^III^**, **C^I^**, **E^I^**, **F^III^**, **F^IV^**, **G^II^**), and inferior (**A^IV^**, **B^II^**, **D^II^**, **G^III^**) views. **A-A^IV^**, RSU DGE 2020 RO MP-10; **B-B^II^**, RSU DGE 2020 RO MP-11; **C-C^I^**, RSU DGE 2020 RO MP-21; **D-D^II^**, RSU DGE 2021 RO MP-4; **E-E^I^**, RSU DGE 2021 RO MP-6; **F-F^IV^**, RSU DGE 2021 RO MP-11; **G-G^III^**, RSU DGE 2021 RO MP-13. Scale bars equal 5 mm.

(Selected synonyms)

p.“Dentem seu palatum piscis Ostracionis”: Brückmann, 1752:116, pl. 5, fig. 4 (non fig. 3), pl. 6, fig. 4 (ipsum dentem exemtum petrefactum, p. 120).

p.† *Ptychodus decurrens* Agass.: Agassiz, 1838:pl. 25b (atlas vol. III), fig. 1, 2, 6–8 (non figs. 3–5).

*Ptychodus decurrens* Agass.: Agassiz, 1839a:154 (vol. 3).

p.*Ptychodus polygyrus* Agass.: Agassiz, 1839b:pl. 25 (atlas vol. III), fig. 9 (non figs. 4–8, 10, 11).

*P*. *decurrens*: Woodward 1887:123, pl. 10, figs. 2–10, 13.

*Ptychodus decurrens*: Woodward, 1904:133, text fig. on p. 134, pl. 15, figs. 1–5.

*Ptychodus decurrens* Agassiz: Woodward, 1912: 239; text fig. 70, 71, 76, 77, pl. 51, 52 (non syn.).

*Ptychodus decurrens* Agassiz L. 1835: Herman, 1977:49, text fig. on p. 52, pl. 2, fig. 1 (non syn.).

*Ptychodus decurrens* Agassiz, 1835: Muller, 2008:62, pl. 1, 2. p.*Ptychodus* sp.: Hoffman, 2016:743, figs. 3.7-9, 14 (non figs. 3.1-6, 7-13).

*Ptychodus decurrens*: Biriukov, 2018:36, pl. 2, Fig. 2a, b.

*Ptychodus decurrens* Agassiz 1835: Hamm, 2020a:26, figs. 37–48 (non syn.).

#### Diagnosis

See Hamm (2020a:26).

#### Species Stratigraphic Range

*Ptychodus decurrens* is a well-known species from the late Cenomanian–middle Turonian of numerous localities around the world, but it has been also rarely reported and figured from Albian deposits of Europe and North America (e.g., Priem, 1912; Welton and Farish, 1993).

#### Referred Material

Eight isolated teeth (RSU DGE 2020 RO MP-10, RSU DGE 2020 RO MP-11, RSU DGE 2020 RO MP-21, RSU DGE 2021 RO MP-4, RSU DGE 2021 RO MP-6, RSU DGE 2021 RO MP-11, RSU DGE 2021 RO MP-13, and KP NVF 19956/16) belonging to the fossil collections of the Ryazan State University and of State Darwin Museum in Moscow.

#### Description

RSU DGE 2020 RO MP-10 (Fig. 5A–A^IV^) is an isolated, polygonal (almost rectangular) tooth with an asymmetrical occlusal outline. In occlusal view (Fig. 5A), the right dental edge is almost straight and slightly depressed, whereas the left one is curved and enlarged posteriorly. The posterior sulcus is shallow and wide. The anterior protuberance is well developed. The crown is largely covered by seven thin, parallel ridges, which branch at their ends and reach the lateral crown margins (see Fig. 5A). Fine granulations without a diagnostic pattern cover the anterior marginal area. In posterior view (Fig. 5A^II^), the crown is gently raised at its center. The thick, bilobate root exhibits a deep antero-posterior sulcus; although the right lobe is more developed and wider than the other. In lateral view (Fig. 5A^III^), the raised portion of the occlusal surface has a posterior outline perpendicular to the crown base; the anterior outline of the crown is inclined instead. The crown juts out from the root on all sides. The lateral outline of the root is straight posteriorly and tilted back on the anterior side (see Fig. 5A^III^). Numerous foramina are placed along the crown/root boundary. In inferior view (Fig. 5A^IV^), the root is rectangular. In occlusal view (Fig. 5B), RSU DGE 2020 RO MP-11 exhibits a polygonal, asymmetrical outline. The crown has a deep posterior sulcus and a slight anterior protuberance. The right marginal area is more developed than the left one. Eight to nine thin, parallel ridges cross transversally on the occlusal surface, branching and reaching the lateral tooth edges (see Fig. 5B). The anterior marginal area is poorly developed and covered by fine granulation. In posterior view (Fig. 5B^I^), the left side of the occlusal crown surface is gently raised. The root is bilobate with a shallow antero-posterior sulcus. In the inferior view (Fig. 5B^II^), the root outline follows the shape of the crown. RSU DGE 2020 RO MP-21 (Fig. 5C, C^I^) exhibits an almost triangular outline with the anterior tooth edge narrow and merged to the right one in a single margin curved posteriorly; the left side is instead slightly convex. The posterior sulcus is shallow. In occlusal view, 8–9 thin, parallel ridges cross the dental crown and reach the lateral tooth edge with branched ends. A fine granulation lacking in diagnostic patterns covers the anterior marginal area of RSU DGE 2020 RO MP-21 (see Fig. 5C). In lateral view (Fig. 5C^I^), the posterior outline of both the crown and root is straight, whereas their anterior outline is tilted. RSU DGE 2021 RO MP-4 (Fig. 5D–D^II^) is quite similar to RSU DGE 2020 RO MP-21 (Fig. 5C, C^I^). In RSU DGE 2021 RO MP-4 the left side is markedly curved posteriorly, whereas the right one is slightly convex; six transversal ridges cover the occlusal surface (see Fig. 5D). In occlusal view (Fig. 5E), RSU DGE 2021 RO MP-6 has a rectangular crown crossed by nine thin, transversal ridges; both the posterior sulcus and anterior protuberance are almost absent. The occlusal ridges branch and thin at their ends reaching the lateral tooth margins. In lateral view (Fig. 5E^I^), RSU DGE 2021 RO MP-6 has a crown with convex surface and straight posterior outline, whereas its anterior margin is tilted downwards; the root is not preserved. RSU DGE 2020 RO MP-11 (Fig. 5F–F^IV^), RSU DGE 2021 RO MP-4 (Fig. 5D–D^II^), and RSU DGE 2021 RO MP-6 (Fig. 5E, E^I^) exhibit depressed areas on the side of their lateral edges. Although the posterior side of RSU DGE 2021 RO MP-11 is partially damaged on the left, its crown is almost rectangular and slightly asymmetrical; both anterior protuberance and posterior sulcus are wide and not marked (see Fig. 5F). Nine to 10 thin, transversal ridges cross the occlusal surface branching on the marginal areas and reaching the lateral tooth edges (see Fig. 5F). Fine granulation covers the rest of the crown without a diagnostic pattern. In anterior (Fig. 5F^I^) and posterior (Fig. 5F^II^) views, the crown of RSU DGE 2021 RO MP-11 is markedly raised and bulged at the center with lateral side externally tilted. In lateral view (Fig. 5F^III^, F^IV^), the anterior side of the crown is inclined downwards. The dental root is missing. RSU DGE 2021 RO MP-13 has a rectangular outline with shallow posterior sulcus and wide, rounded anterior protuberance (see Fig. 5G). The lateral surface of the left side shows two depressed areas (posterior and anterior), whereas the right one has a quite rounded outline. Nine to 10 thin ridges transversally cross the occlusal tooth surface, branching at their ends and reaching the lateral crown edges. Fine granulation and wrinkles characterize the anterior protuberance (see Fig. 5G). In posterior view (Fig. 5G^I^), the dental crown is bulged in the center with inclined lateral marginal areas; the root is bilobate with a shallow antero-posterior sulcus. In lateral view (Fig. 5G^II^), the anterior crown outline is tilted downwards; the tooth root exhibits a posterior side perpendicular to the crown base, whereas its anterior outline is inclined. In inferior view (Fig. 5G^IV^), the root follows the rectangular outline of the crown. Irregular, unbranched, and whitish traces are on occlusal and posterior sides of RSU DGE 2021 RO MP-11 (see black arrows in Fig. 5F-F^III^) and RSU DGE 2021 RO MP-13 (see black arrows in Fig. 5G–G^II^). KP NVF 19956/16 and RSU DGE 2021 RO MP-6 (Fig. 5E, EI) are similar to each other and exhibit only the dental crown with a raised central area. Nine thin, transversal ridges cross the entire occlusal surface, reaching the marginal area. The ridges branch at their ends at the lateral tooth edges and are slightly abraded at the center of the tooth crown. Fine wrinkles cross the anterior protuberance.

#### Remarks

An optimal preservation characterizes most of the material assigned here to *Ptychodus decurrens*, which exhibit complete crowns and roots with few exceptions (e.g., RSU DGE 2021 RO MP-6 and RSU DGE 2021 RO MP-11). No occlusal ‘articular facets’ are recognizable on the tooth edges of the specimens of *P*. *decurrens* documented herein. The asymmetrical outline of all the specimens (RSU DGE 2020 RO MP-10, Fig. 5A–A^IV^; RSU DGE 2020 RO MP-11, Fig. 5B–B^II^; RSU DGE 2020 RO MP-21, Fig. 5C, C^I^; RSU DGE 2021 RO MP-4, Fig. 5D–D^II^; KP NVF 19956/16) indicates their placement within the lateral tooth rows of the dentition. In particular, RSU DGE 2020 RO MP-10 was placed close to the symphyseal row, whereas RSU DGE 2020 RO MP-11 and RSU DGE 2020 RO MP-21 were arranged in the distal-most rows. RSU DGE 2020 RO MP-10, RSU DGE 2020 RO MP-11, and RSU DGE 2021 RO MP-4 probably belong to the lower dental plate, having a raised crown. Tooth abrasions have been rarely reported for *P*. *decurrens* (e.g., Woodward, 1887; Amalfitano et al., 2020). Among the teeth assigned to this *P*. *decurrens*, only KP NVF 19956/16 exhibits dental wear patterns. *Ptychodus decurrens* is an un-cusped species characterized by juxtaposed dentitions composed by teeth with irregular occlusal outline and lateral ‘facets’ (including symphyseals; e.g., Woodward, 1887:pl. X, figs. 1, 3, 10). The slightly depressed imprints (‘facets’) characterizing the lateral side of the crown edges in RSU DGE 2020 RO MP-10, RSU DGE 2021 RO MP-4, and RSU DGE 2021 RO MP-6 (see Fig. 5E^I^) were probably useful in interlocking with adjacent teeth (juxtaposition). Commonly, symphyseal teeth in *Ptychodus* show a symmetrical outline (e.g., Shimada, 2012; Amadori et al., 2020a; Hamm, 2020a). Although RSU DGE 2021 RO MP-11 (Fig. 5F) and RSU DGE 2021 RO MP-13 (Fig. 5G) have a slightly asymmetrical, bulged crown, they likely belong to the symphyseal row of the lower dental plate.

### †Ptychodus Latissimus Agassiz, 1835 (Fig. 6)

(Selected synonyms)

p.“Teeth allied to *Diodon*”: Mantell, 1822:231, pl. 32, fig. 19 (non figs. 17, 18, 20, 21, 23–25, 27, 29).

†*Ptychodus latissimus* Ag.: Agassiz, 1835:54 (Feuilleton additionnel).

p.*Ptychodus latissimus* Agass.: Agassiz, 1837:pl. 25a (atlas vol. III), figs. 5, 6 (non figs. 1–4, 7, 8).

*Pt. latissimus* Agass.: Agassiz, 1838:pl. 25b (atlas vol. III), figs. 24-26.

*Ptychodus*: Vodorezov and Solonin, 2017:140, fig. 2 (above).

v*Ptychodus latissimus* Agassiz 1835: Amadori et al., 2020a:4, fig. 3 (cum syn.).

p*Ptychodus latissimus* Agassiz 1835: Hamm, 2020a:46, figs. 69–71 (non figs. 67, 68) (non syn.).

*Ptychodus latissimus* Agassiz 1835: Amadori et al., 2020b:7, figs. 1, 2, 4, 6–12.

*Ptychodus latissimus* Agassiz 1835: Hamm, 2020a:46, figs. 67–71.

#### Diagnosis

See Amadori et al. (2020a:6).

#### Species Stratigraphic Range

The temporal range currently documented for *Ptychodus latissimus* is late Turonian–early Coniacian (see Hamm 2020a:fig. 100).

#### Referred Material

A single tooth (SS106-1), housed in the collection of the Sasovo secondary school n. 106.

#### Description

**Figure 6:**
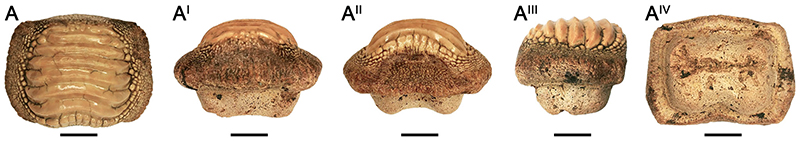
Tooth (**A-A^IV^**, SS106-1) of *Ptychodus latissimus* Agassiz, 1835, from the Upper Cretaceous of Ryazan Oblast (western Russia) in occlusal (**A**), anterior (**A^I^**), posterior (**A^II^**), lateral (**A^III^**), and inferior (**A^IV^**) views. Scale bars equal 10 mm.

Specimen SS106-1 (Fig. 6A–A^IV^) is an isolated tooth characterized by an almost symmetrical outline. Both anterior protuberance and posterior sulcus are poorly developed. In occlusal view (Fig. 6A), eight thick, sharp ridges cross the dental surface. The anterior-most and posterior-most ridges are interrupted and finer than the others. All ridges terminate abruptly without reaching the lateral tooth edges. The anterior-most ridges are slightly damaged, but overall dental wear is absent or very limited. Large granular bumps surround the crested area, whereas thin granules prolong the ends of the ridges. Nevertheless, the crested area is clearly separated from the marginal ornamentations. The marginal area is covered by coarse granulations without diagnostic patterns. In anterior view (Fig. 6A^I^), the center of the thick crown is bulged. The pre-served tooth root is bilobate with a shallow antero-posterior sulcus. The lateral root sides slightly converge inwards. In inferior view (Fig. 6A^IV^), the tooth crown overhangs the root on all sides. The root is rectangular and partially damaged.

#### Remarks

The material presented herein is well preserved; both crown and root of SS106-1 (Fig. 6A-A^IV^) are almost complete. Moreover, this large tooth was probably situated within the lateral area of the dentition and close to the symphyseal row. The occlusal ridges are only slightly damaged and the observed scratches do not conform to the typical dental wear commonly related to the prey processing in this species (also see Amadori et al., 2020a, 2020b). In addition, Vodorezov and Solonin (2017:fig. 2) described and figured an isolated tooth RSU DGE 2017 RO MP-34 from the Malyy Prolom quarry; the specimen is currently lost. RSU DGE 2017 RO MP-34 has an asymmetrical outline and eight ridges transversally arranged on the center of the occlusal surface. The ridges are abruptly interrupted at their ends and well separated from the marginal ornamentation. Coarse granules cover the marginal area. Although only one image (latero-occlusal view; see Vodorezov and Solonin, 2017:fig. 2) is currently available for RSU DGE 2017 RO MP-34, its asymmetrical outline would indicate its arrangement along one of the lateral tooth rows; moreover, the small size and thinned ridges of this tooth indicate its distal position within the dentition.

### †Ptychodus Mammillaris Agassiz, 1835 (Fig. 7)

(Selected synonyms)

p.“Teeth allied to *Diodon*”: Mantell, 1822:231, pl. 32, fig. 20 (non figs. 17–19, 21, 23–25, 27, 29).

†*Ptychodus mammillaris* Ag.: Agassiz,1835:54 (Feuilletton additionnel).

*Pt. mammillaris* Agass.: Agassiz, 1838:pl. 25b (atlas vol. III), figs. 11–20.

*Ptychodus altior* Agass.: Agassiz, 1838:pl. 25b (atlas vol. III), figs. 9, 10.

p.*Ptychodus decurrens* Agass.: Agassiz, 1838:pl. 25b (atlas vol. III), Fig. 3 (non figs. 1, 2, 4–8).

p.*Ptychodus polygyrus* Agass.: Agassiz, 1839:pl. 25 (atlas vol. III), figs. 5, 6 (non figs. 4, 7–11).

*Ptychodus mammillaris* Agass.: Agassiz, 1839:151 (vol. III).

*Ptychodus altior* Agass.: Agassiz, 1839:155 (vol. III).

*Ptychodus mammillaris* Agassiz: Woodward, 1912:230, text fig. 72, pl. 47, figs. 13-27 (non syn.).

*Ptychodus mammillaris* Agassiz 1835: Hamm, 2020a:31, figs. 52-60 (non syn.).

#### Diagnosis

See Hamm (2020a:33).

#### Species Stratigraphic Range

Currently, the stratigraphic distribution for *Ptychodus mammillaris* is middle Turonian-middle Coniacian (see Hamm 2020a:fig. 100).

#### Referred Material

Seven isolated teeth (RSU DGE 2020 RO MP-6, RSU DGE 2020 RO MP-12, RSU DGE 2021 RO MP-2, RSU DGE 2021 RO MP-14, RSU DGE 2021 RO MP-20, SS106-4, and SS106-7) belonging to the collections of the Ryazan State University and the Sasovo secondary school n. 106.

#### Description

**Figure 7:**
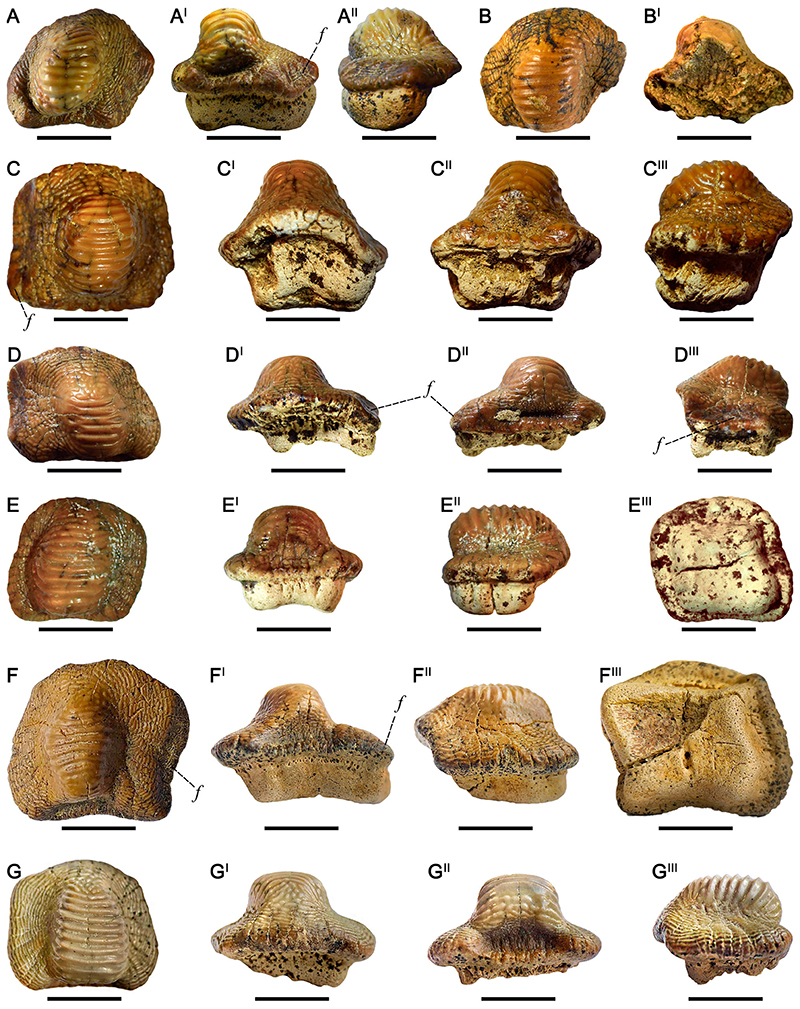
Teeth of *Ptychodus mammillaris* Agassiz, 1835, from the Upper Cretaceous of Ryazan Oblast (western Russia) in occlusal (**A**, **B**, **C**, **D**, **E**, **F**, **G**), anterior (**C^I^**, **D^I^**, **G^I^**), posterior (**A^I^**, **B^I^**, **C^II^**, **D^II^**, **E^I^**, **F^I^**, **G^II^**), lateral (**A^III^**, **C^III^**, **D^III^**, **E^II^**, **F^II^**, **G^III^**), and inferior (**E^III^**, **F^III^**) views. **A–A^III^**, RSU DGE 2020 RO MP-6; **B–B^I^**, RSU DGE 2020 RO MP-12; **C–C^III^**, RSU DGE 2021 RO MP-2; **D–D^III^**, RSU DGE 2021 RO MP-14; **E–E^III^**, RSU DGE 2021 RO MP-20; **F, F^III^**, SS106-4; **G–G^III^**, SS106-7. Scale bars equal 10 mm.

RSU DGE 2020 RO MP-6 (Fig. 7A–A^IV^) and RSU DGE 2020 RO MP-12 (Fig. 7B, B^I^) have quadrangular crowns similar to each other with asymmetrical outline and a single knob-like cusp moved on the left side. In both teeth, the anterior protuberance is rounded and the left dental edge curved backwards. Their cusps are crossed by 9–10 thin ridges, which are well-separated by marginal ornamentations. Their marginal areas are well developed and entirely covered by thin, concentric wrinkles. In occlusal view (Fig. 7A), the right tooth edges of RSU DGE 2020 RO MP-6 are characterized by a lowered margin (see also ‘*f*’ in Fig. 7A^I^); the posterior sulcus is deep. In lateral view (Fig. 7A^II^), the cusp of RSU DGE 2020 RO MP-6 shows an anterior outline tilted downwards. RSU DGE 2020 RO MP-6 exhibits slight abrasion on the right lateral surface of its cusp apex (see Fig. 7A, A^II^). The right side of RSU DGE 2020 RO MP-12 (Fig. 7B, B^I^) is broken and the root is missing. The tooth wear in RSU DGE 2020 RO MP-12 (Fig. 7B) involves the entire cusp apex. In occlusal view, RSU DGE 2021 RO MP-2 (Fig. 7C–C^III^) has an almost quadrangular, cuspidate crown with a wide posterior sulcus and a rounded and poorly developed anterior protuberance. The left dental margin of RSU DGE 2021 RO MP-2 is depressed (see ‘*f*’ in Fig. 7C). Its cusps exhibit approximately 10 thin ridges; concentric wrinkles characterize the marginal areas (see Fig. 7C). In posterior view (Fig. 7C^II^), RSU DGE 2021 RO MP-2 shows a knob-like cusp moved on the right side of its crown. The crown of RSU DGE 2021 RO MP-2 juts out from the root on all sides with the lateral edges curved downwards in anterior view (Fig. 7C^I^); the root is bilobate with a shallow antero-posterior sulcus and lateral sides tilted inwards. In lateral view (Fig. 7C^III^), RSU DGE 2021 RO MP-2 has a posterior outline perpendicular to the crown base and an anterior side tilted forward; the root exhibits an anterior side inclined posteriorly (see also Fig. 7C^III^). In occlusal view, RSU DGE 2021 RO MP-14 (Fig. 7D–D^III^) has a nearly square outline with a marked anterior protuberance and a wide and deep posterior sulcus. In RSU DGE 2021 RO MP-14, the left lateral edge is concave and slightly depressed, whereas the right one is convex and curved posteriorly (see also ‘*f*’ in Fig. 7D^I^–D^III^). The occlusal surface exhibits a narrow cusp with a knob-like apex covered by six thin, transversal ridges that are confined to the cusp surface (see Fig. 7D). Thin, concentric granulations characterize the entire marginal area. In posterior view (Fig. 7D^II^), the lateral edges of the crown are tilted downwards, whereas the cusp is high and rounded. In lateral view (Fig. 7D^III^), the crown base is flat; the cusp exhibits a straight posterior side, whereas the anterior outline is inclined reaching the tooth margin. The tooth root is almost completely missing. RSU DGE 2021 RO MP-20 (Fig. 7E–E^IV^) has quadrangular and slightly asymmetrical occlusal outline with arounded anterior protuberance and a shallow posterior sulcus; moreover, the left crown edge curves posteriorly. The occlusal surface has a large cusp with a knob-like apex. Nine thin, transversal ridges cover the cusp surface (see Fig. 7E). In posterior view (Fig. 7E^I^), the crown has both lateral edges curved downwards; the dental root is bilobate and it exhibits a deep antero-posterior sulcus. In lateral view (Fig. 7E^II^), the cusp is low and almost flat and the ridges term abruptly at the base of the cusp. The posterior tooth side is perpendicular to the crown base, whereas the anterior crown outline is inclined downwards. The marginal area of RSU DGE 2021 RO MP-20 is thin and flat. In inferior view (Fig. 7E^IV^), the crown juts out from the square root on all sides. SS106-4 (Fig. 7F–F^III^) and SS106-7 (Fig. 7G–G^III^) share general tooth features and occlusal ornamentations (both ridges and granulations) with RSU DGE 2021 RO MP-14 (Fig. 7D–D^III^). The right edges of SS106-4 are concave, whereas the left one is curved posteriorly (see Fig. 7F). The right tooth edge is slightly depressed at its external margin. The apical surface of the knob-like cusp exhibits nine thin, transversal ridges. The crown juts out from a bilobate root on all sides. In posterior view (Fig. 7F^I^), the left lobe of the dental root is the smallest and its external side is inclined towards the center of the root. In inferior view (Fig. 7F^III^), the tooth root is square and follows the crown occlusal outline. In occlusal view (Fig. 7G), the crown of SS106-7 is almost symmetrical with a straight left tooth edge and a rounded outline on the opposite side; the tooth cusp is narrow anteriorly. In anterior view (Fig. 7G^I^), the cusp is almost flat and the bilobate root is broken on the left. RSU DGE 2021 RO MP-14, SS106-4, and SS106-7 exhibit slight wear traces on the occlusal ridges.

#### Remarks

RSU DGE 2020 RO MP-6 (Fig. 7A–A^IV^) and RSU DGE 2020 RO MP-12 (Fig. 7B, B^I^) were placed along distal tooth rows within the left hemiarch, whereas RSU DGE 2021 RO MP-2 (Fig. 7C–C^III^) likely was an antero-lateral teeth probably arranged close to the symphyseal row in the right portion of the dentition. The asymmetrical occlusal outline of RSU DGE 2021 RO MP-14 (Fig. 7D–D^III^), RSU DGE 2021 RO MP-20 (Fig. 7E–E^III^), SS106-4 (Fig. 7F–F^III^), and SS106-7 (Fig. 7G–G^III^) indicate they belong to lateral dental rows (e.g., L1–L4 in Hamm, 2020a:fig. 57). RSU DGE 2021 RO MP-14 and SS106-7 were probably arranged within the right hemiarch of the dentition, whereas RSU DGE 2021 RO MP-20 and SS106-4 in the left one. The squared occlusal outline of RSU DGE 2021 RO MP-20, SS106-4, and SS106-7 excludes their distal position within the tooth plate. Dignathic heterodonty in *P*. *mammillaris* is still poorly known. The attribution of these teeth to either the maxillary or mandibular dentition is therefore tentative until further studies on articulated plates of *P*. *mammillaris* can be made. The depressed imprints (‘articular facets’) on the occlusal surface of the medial tooth edges of RSU DGE 2021 RO MP-14 (Fig. 7D^I^–D^III^) and SS106-4 (Fig. 7F, F^I^) indicate that this taxon was characterized by imbricated dentitions, as depicted in the artificially rearranged lower plate provided by Hamm (2020:fig. 57).

### † ptychodus marginalis (sensu Hamm, 2020a) (Fig. 8)

#### Diagnosis

See Hamm (2020a:24).

#### Species Stratigraphic Range

Currently, the stratigraphic distribution for *Ptychodus marginalis* is late Cenomanian–middle Turonian (see Hamm, 2020a:fig. 100).

#### Referred Material

Two isolated teeth (SS106-2 and SS106-3) belonging to the fossil collection of the Sasovo secondary school n. 106.

#### Description

**Figure 8:**
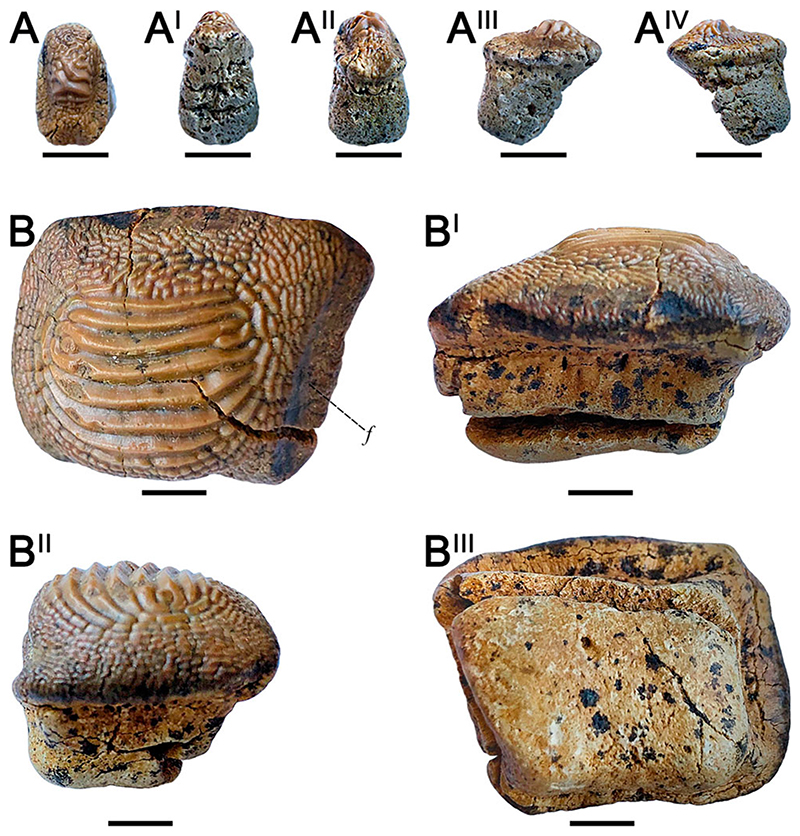
Teeth here assigned to *Ptychodus marginalis* sensu Hamm (2020a) from the Upper Cretaceous of Ryazan Oblast (western Russia) in occlusal (**A**, **B**), anterior (**A^I^**, **B^I^**), posterior (**A^II^**), lateral (**A^III^**, **A^IV^**, **B^II^**), and inferior (**B^III^**) views. **A-A^IV^**, SS106-2; **B-B^III^**, SS106-3. Scale bars equal 5 mm.

Specimen SS106-2 (Fig. 8A–A^IV^) is antero-posteriorly elongated in general shape. In occlusal view (Fig. 8A), its crown exhibits an almost rectangular outline with a rounded anterior margin; the posterior side is larger than the opposite one with a shallow posterior sulcus. The mesio-distally compressed occlusal surface shows three short and irregular ridges with anastomoses (no concentric ‘loops’); thin wrinkles cover the anterior marginal area, whereas fine granulations characterize the posterior occlusal area. In anterior and posterior views (Fig. 8A^I^, A^II^), the crown of SS106-2 is thin and narrow and the lateral edges are characterized by depressed and tilted areas. The root is thick and monolobate with a rounded and large end; both lateral root sides are inclined externally (see also Fig. 8A^I^, A^II^). The crown base juts out from the root on all the sides. In lateral view (see Fig. 8A^III^, A^IV^), the crown base is flat and the crested area is raised; the root is markedly tilted posteriorly. The dental root is slightly damaged anteriorly. In occlusal view (Fig. 8B–B^III^), SS106-3 has an asymmetrical and trapezoidal outline with the anterior side of the crown markedly wider than the posterior one. Both anterior protuberance and posterior sulcus are poorly developed. The right tooth edge is rounded and convex, whereas the left one is slightly concave. Eight ridges characterized the center of the crown occlusal surface; the ridge ends become thinner without reaching the tooth edges (see Fig. 8B). The ridges curve anteriorly and gradually blend with fine concentric wrinkles and granulations, covering the entire marginal area. The anterior marginal area is well developed, whereas the posterior one is almost absent. A depressed area is recognizable along the left marginal area (see *f* in Fig. 8B). Although the occlusal ridges are slightly damaged, traces of dental wear are clearly observable on the left side of the crested area (see Fig. 8B, B^II^). In anterior view (Fig. 8B^I^), the lateral crown edges are inclined downwards, whereas the crested area is gently raised and moved on the right side. The dental root is thick and bilobate with both lateral sides inclined towards the center of the tooth; the antero-posterior sulcus is shallow (see Fig. 8B^I^). The inferior portion of SS106-3 is cracked anteriorly. In lateral view (Fig. 8B^II^), the dental crown is slightly raised with a posterior outline parallel to the tooth base and the anterior side curved downwards. The posterior side of the tooth root is straight, whereas the anterior one is tilted back (see also Fig. 8B^II^). In inferior view (Fig. 8B^III^), the crown protrudes from the root on all sides; the root follows the polygonal outline of the dental crown.

#### Remarks

The general, antero-posteriorly elongated shape of the crown, together the monolobate root, described for SS106-2 (Fig. 8A–A^IV^) is typical of upper symphyseal teeth in un-cuspidate *Ptychodus* (see also Woodward, 1887, 1912; Amadori et al., 2020a; Hamm, 2020a). Moreover, upper symphyseal teeth of *P*. *marginalis* almost identical to that presented herein have been previously documented by Hamm (2020a:figs. 36.1–36.2). Nevertheless, upper symphyseals of *P*. *polygyrus* are also similar to those of *P*. *marginalis* (see Woodward, 1912:pl. 48, fig. 13O^I^ and pl. 49, fig. 1O^I^). The depressed and tilted margins (Fig. 8A^I^, A^II^) on the lateral edges of SS106-2 were likely useful for imbrication with adjacent dental rows (‘L2’, ‘R2’ in Hamm, 2020a:Fig. 32) below the occlusal plane, as already documented for other un-cuspidate taxa (e.g., *P*. *mediterraneus*; see Amadori et al., 2020a:fig. 20). Although SS106-3 (Fig. 8B–B^III^) shows occlusal ornamentations similar to those previously described for *P*. *polygyrus* (e.g., Amadori et al., 2020a; Hamm, 2020a), the concentric pattern of the marginal ornamentation in SS106-3 is typical of *P*. *marginalis* (see Amadori et al., 2020a; Hamm, 2020a). *Ptychodus marginalis* is an un-cuspidate taxon with both ridges and marginal ornamentation that is concentrically arranged (see ‘Emended diagnosis’ section in Hamm, 2020a:24). In addition, Amadori et al. (2020a) recently have described the main morphological differences between various un-cuspidate species (e.g., *P*. *latissimus*, *P*. *marginalis*, and *P*. *polygyrus*; see also Hamm, 2010, 2020a). Specimen SS106-3 probably was placed along one of the left lateral rows of the dentition. The depressed imprint on the right dental edge of SS106-3 indicates that the distal margin of the adjacent tooth overlapped (imbrication) the mesial one in SS106-3 (right side in Fig. 8B). Imbricated dentitions have been previously described for other un-cuspidate taxa of *Ptychodus* (e.g., *P*. *latissimus* and *P*. *mediterraneus;* see Amadori et al., 2020a, 2020b). Moreover, the inclined sides of the dental root in SS106-3 (see Fig. 8B^II^) would facilitate the tooth arrangement on the jaws while maintaining an antero-posterior curvature of the grinding plate.

### †Ptychodus cf. †P. Mediterraneus Canavari, 1916 (Fig. 9)

#### Diagnosis

See Amadori et al. (2020a:9).

#### Species Stratigraphic Range

Currently, the stratigraphic distribution for *Ptychodus mediterraneus* is Turonian–Coniacian (see Amadori et al., 2020a).

#### Referred Material

A single isolated tooth RSU DGE 2021 RO MP-10 belonging to the fossil collections of the Ryazan State University.

#### Description

**Figure 9:**
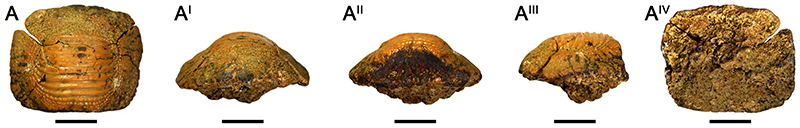
Tooth (**A-A^IV^**, RSU DGE 2021 RO MP-10) here assigned to *Ptychodus* sp. cf. *P*. *mediterraneus* Canavari, 1916, from the Upper Cretaceous of Ryazan Oblast (western Russia) in occlusal (**A**), anterior (**A^I^**), posterior (**A^II^**), lateral (**A^III^**), and inferior (**A^IV^**) views. Scale bars equal 10 mm.

Specimen RSU DGE 2021 RO MP-10 exhibits a poorly preserved crown and it lacks a root. In occlusal view (Fig. 9A–A^IV^), RSU DGE 2021 RO MP-10 has a rectangular and symmetrical outline with a wide and rounded anterior protuberance and a shallow posterior sulcus. Ten to 11 thick ridges cross the tooth crown, but fail to reach the lateral dental edges; large bumps surround the crested area. The ridges curve anteriorly and thin at their ends; a coarse granulation covers the marginal areas. In anterior and posterior views (Fig. 9A^I^, A^II^), the occlusal surface is convex and rounded with later edges slightly inclined downwards. The crested area of RSU DGE 2021 RO MP-10 is markedly abraded (see Fig. 9A).

#### Remarks

The tooth shape and occlusal ornamentations of RSU DGE 2021 RO MP-10 (Fig. 9A–A^IV^) resemble those described for symphyseal teeth of *Ptychodus mediterraneus* (e.g., Amadori et al., 2020a:fig. 17I, J). Moreover, crested and marginal areas are clearly distinguishable from each other in RSU DGE 2021 RO MP-10 (Fig. 9A). The irregular wrinkles typically documented on the anterior protuberance of *P*. *mediterraneus* are barely recognizable on RSU DGE 2021 RO MP-10 due to the poorly preserved anterior marginal ornamentation. However, the absence of loops, anastomoses and concentric ridge patterns make unlikely its attribution to other un-cuspidate species, such as *P*. *marginalis* and *P*. *polygyrus*. A close affinity between RSU DGE 2021 RO MP-10 and *P*. *mediterraneus* is therefore hypothesized herein based on their crown general features.

### †Ptychodus Polygyrus Agassiz, 1835 (Fig. 10)

(Selected synonyms)

p.‘Teeth allied to *Diodon*’: Mantell, 1822:231, pl. 32, figs. 23, 24 (non figs. 17–21, 25, 27, 29).

†*Ptychodus polygyrus* Ag.: Agassiz, 1835:54 (Feuilleton additionnel).

p.*Pt. polygyrus* Agass.: Agassiz, 1838:pl. 25b (atlas vol. III), figs. 22, 23 (non figs. 21).

p.*Ptychodus polygyrus* Agass.: Agassiz, 1839:pl. 25 (atlas vol. III), figs. 4, 7, 8, 10, 11 (non figs. 5, 6, 9).

p.*Ptychodus latissimus* Agassiz: Woodward, 1912:235, pl. 50, figs. 1–3, 9, 10, 13–16 (non figs. 4–8, 11, 12).

p.*Ptychodus polygyrus* Agassiz: Woodward, 1912:232, pl. 48, figs. 12, 15, 16, pl. 49 (non pl. 48, figs. 13, 14).

*Ptychodus* sp.: Aleksandrovich, 2019:265, fig. 1.

v*Ptychodus polygyrus* Agassiz 1835: Amadori, Amalfitano, Giusberti, Fornaciari, Carnevale, & Kriwet 2020a:7, fig. 4 (cum syn.).

*Ptychodus polygyrus* Agassiz 1835: Hamm, 2020a:65, figs. 90–94, 95 (right) (non syn.).

v*P*. *marginalis*: Solonin, Shell, Vodorezov, & Niedźwiedzki, 2020:fig. 5.2a, b.

### Diagnosis

See Amadori et al. (2020a:8).

### Species Stratigraphic Range

*Ptychodus polygyrus* is well-known for Santonian to Campanin (see Hamm, 2020a:fig. 100).

### Referred Material

Three isolated teeth (RSU DGE 2018 RO MP-41, RSU DGE 2021 RO MP-19, and KP NVF 19956/215) belonging to the collections of the Ryazan State University and of State Darwin Museum in Moscow.

### Description

**Figure 10:**
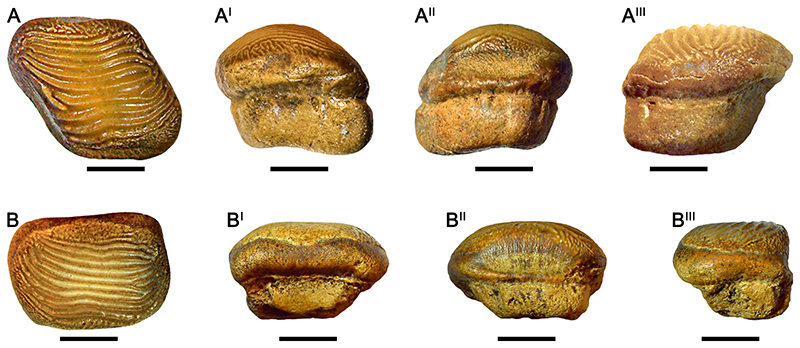
Teeth of *Ptychodus polygyrus* Agassiz, 1835, from the Upper Cretaceous of Ryazan Oblast (western Russia) in occlusal (**A**, **B**), anterior (**A^I^**, **B^I^**), posterior (**A^II^**, **B^II^**), and lateral (**A^III^**, **B^III^**) views. **A-A^III^**, RSU DGE 2018 RO MP-41; **B-B^111^**, RSU DGE 2021 RO MP-19. Scale bars equal 5 mm.

RSU DGE 2018 RO MP-41 (Fig. 10A–A^III^) and KP NVF 19956/215 exhibit rectangular, asymmetrical tooth crowns almost completely covered by 10–11 thick transversal ridges. Both anterior protuberance and posterior sulcus are not marked. The right tooth edges of KP NVF 19956/215 are broken. In occlusal view (Fig. 10A), the ridges curve anteriorly reaching the lateral dental edges; anastomoses and loops are also recognizable at the right ends of the ridges. Coarse granulations without a diagnostic pattern cover the anterior marginal area. In anterior view (Fig. 10A^II^), both teeth have massive, slightly raised tooth crowns; the roots are bilobate and thick with a shallow antero-posterior sulcus. In lateral view (Fig. 10A^III^), the outline of the occlusal surface is inclined anteriorly; the root has a posterior side perpendicular to the crown base, whereas the anterior outline is tilted posteriorly. The crown of RSU DGE 2018 RO MP-41 has a slight lateral depression on the side of the left edge (see Fig. 10A, A^II^). RSU DGE 2021 RO MP-19 (Fig. 10B–B^III^) has a rectangular crown with both posterior sulcus and anterior protuberance poorly developed. The right crown edge is almost straight, whereas the left one is curved. Ten transverse, thin ridges cross the occlusal surface, branching at their ends and reaching the lateral tooth margins (see Fig. 10B). Fine granulation covers the anterior marginal area without a diagnostic pattern. In posterior view (Fig. B^II^), RSU DGE 2021 RO MP-19 exhibits a flat crown. The root has a shallow antero-posterior sulcus and the outline of the left side is markedly inclined. In lateral view (Fig. B^III^), the anterior sides of both crown and root are tilted. The posterior outline of the tooth is instead straight.

### Remarks

The bilateral asymmetry and the raised occlusal surface shown by both specimens (RSU DGE 2018 RO MP-41 and KP NVF 19956/215) indicate their belonging to the lateral rows of the lower dentition. However, they were probably arranged within opposite sides of the dentition. Based on its asymmetrical outline and its flat crown, RSU DGE 2021 RO MP-19 (Fig. 10B–B^III^) was probably arranged along the lateral rows of the upper dental plate. Both RSU DGE 2018 RO MP-41 and RSU DGE 2021 RO MP-19 are exceptionally well preserved, exhibiting complete crowns and roots. Any ‘articular facet’ is missing on their occlusal marginal area, whereas the left tooth side of RSU DGE 2018 RO MP-41 exhibits a slightly depressed imprint (‘facet’; see Fig. 10A, A^II^). This lateral imprint was probably useful for interlocking with adjacent teeth (juxtaposition). Similar lateral ‘facets’ are also observable in other un-cuspidate species (e.g., *Ptychodus decurrens*; Woodward, 1887; Amadori et al., 2019a; Hamm, 2020a; this paper, see above).

Additional fragmentary and/or abraded teeth (see Fig. S1) are described in the Supplemental Data.

## Discussion

### Taphonomic Remarks

Post-Paleozoic elasmobranchs (sharks, skates, and rays) are well known for their fragmentary record, which is largely composed of isolated teeth with a wide range of sizes and preservational degrees (e.g., Cappetta, 2012; Underwood et al., 2015). Consequently, most studies over the last century focused on articulated material and large teeth in order to obtain higher quality and more reliable information (Underwood et al., 2015). In this regard, ptychodontid sharks are no exception with a huge amount of heavily broken or badly preserved isolated teeth representing their fossil record (Woodward, 1912; D’Erasmo, 1922; Herman, 1977; Cappetta, 2012; Hamm, 2020a). Indeed, much of the material that has been documented for all Ptychodontid genera so far (*Heteroptychodus*, *Ptychodus*, and *Paraptychodus*) consists of isolated teeth, often lacking the root and/or part of the dental crown (e.g., Cappetta, 2012; Hamm, 2020a). Only in recent decades, an increasing amount of isolated material, both unknown and previously collected, has been extensively studied (Underwood et al., 2015).

Most of the shark and ray teeth collected from the Upper Cretaceous of Ryazan Oblast (European Russia) up to now are fragmentary. Only about 8% of the non-ptychodontid teeth are completely preserved, whereas about 50% of tooth crowns are broken and in 92% of the specimens the root is poorly preserved or totally missing. Furthermore, about 80% of the teeth exhibit moderate abrasions on both crowns and roots. Actinopterygian teeth from the same fossil site are mostly damaged, broken and/or worn as well.

Among the isolated teeth of *Ptychodus* from the Upper Cretaceous of Ryazan Oblast (European Russia) that are here examined, only 27% of the specimens are fragmentary, whereas the remaining specimens represent more or less well-preserved tooth crowns (see also Table S1 in Supplemental Data). The teeth of *Ptychodus* examined here therefore show a relatively good state of preservation compared with those usually observed in this group and in other elasmobranch assemblages. Moreover, whitish irregular signs characterize the occlusal surface of RSU DGE 2020 RO MP-14 (see black arrows in Fig. 4E–E^III^), RSU DGE 2020 RO MP-11 (see black arrows in Fig. 5F, F^II^, F^III^), and RSU DGE 2021 RO MP-13 (see black arrows in Fig. 5G–G^II^). Similar traces with rounded extremities and irregular patterns were documented for various isolated shark teeth from the mid-Cretaceous of eastern England and on tooth sets of *Ptychodus* from the Upper Cretaceous of northern Italy (see Amadori et al., 2020a:fig. 18A, C–E, H, I; Underwood et al., 1999:fig. 1b–h). These whitish traces probably result from bioerosion by endolithic organisms and are morphologically close to the fungal borings characterizing the ichnospecies *Mycelites ossifragus* (Roux, 1887; Underwood et al., 1999; Amadori et al., 2020a).

The isolated teeth of *Ptychodus* from the Cenomanian of the Varavinsky Ravine locality (Moscow Oblast) are poorly preserved with only broken tooth crowns and no specimens exhibit the root.

Rare articulated skeletal remains of Mesozoic elasmobranchs are considered to provide detailed morphological information, which is essential for better understanding their evolution and ecology (e.g., Shimada, 2005; Shimada et al., 2009; Cappetta, 2012; Underwood et al., 2015; Collareta et al., 2017; Amalfitano et al., 2017; Amadori et al., 2020a). However, skeletal remains of *Ptychodus* are extremely rare. Nevertheless, the massive sampling and rigorous study of an increasing number of isolated teeth of elasmobranch fishes has led to a tremendous improvement in our knowledge about their diversity and distribution patterns as well as their systematic affinities (Reis, 2005; Cappetta, 2012; Underwood et al., 2015; Bogan et al., 2016; Marramà et al., 2018). Isolated teeth of *Ptychodus* species therefore also have the potential to address various paleobiological topics going far beyond pure taxonomic aspects.

### Tooth Wear and Trophic Ecology

Occupying various trophic levels and representing apex as well as mesopredators with a wide range of diet preferences and multiple feeding strategies (e.g., selective, opportunistic, specialized, or generalized), elasmobranchs are very important to ensure stability in structure and function of marine communities (Cortés, 1999; Baum and Worm, 2009; Myers et al., 2007; Jacobsen and Bennett, 2013; Munroe et al., 2014; Grubbs et al., 2016; Yokoi et al., 2017). Numerous extant elasmobranch groups evolved peculiar adaptations (e.g., molariform teeth arranged in dental plates), which allow them to specialize for exploiting food sources that are unavailable for competitors (niche specialization; see Papastamatiou et al., 2006; Kinney et al., 2011; Vaudo and Heithaus, 2011; Yick et al., 2011; Bornatowski et al., 2014; Ruocco and Lucifora, 2016; Lear et al., 2021; Bazzi et al., 2021). Occlusal wear patterns detected on molariform teeth are useful for inferring specific durophagous specializations and related trophic preferences, as well as investigating tooth renewal patterns, in fossil and extant elasmobranchs (e.g., Woodward, 1912; Niedźwiedzki and Kalina, 2003; Claeson et al., 2010; Cappetta, 2012; Kolmann et al., 2015; Amadori et al. 2019b, 2020a, 2022). Although a voracious predator such as *Ptychodus* probably was occasionally able to feed on a wide range of prey (e.g., bivalves, cephalopods, and small fishes) reaching remarkable size, durophagous specialized taxa were well represented within the genus (see Woodward, 1912; Niedźwiedzki and Kalina, 2003; Cappetta, 2012; Shimada, 2012; Diedrich, 2013; Everhart, 2017; Amadori et al., 2019b, 2020a, 2022; Hamm, 2020a, b).

About half of the isolated teeth of *Ptychodus* identified from the Upper Cretaceous of Malyy Prolom (Ryazan Oblast) exhibit dental wear patterns with various degrees of abrasion related to the procession of shelled prey items. Interestingly, most of the worn teeth (ca. 87%) are assigned to cuspidate species (see Table S1 in Supplemental Data). Therefore, teeth with occlusal cusps might not offer a significant advantage in preventing prey abrasion. However, the loss and subsequent replacement of teeth in *Ptychodus* might not be strictly dependent on the increasing occurrence of damaged and/or not fully functional teeth. The tooth plate might thus progress anteriorly out of the oral cavity leading to the removal and replacement of both worn and intact teeth. In addition, a possible sampling bias could have influenced the result of the preliminary analysis presented here due to the limited number of low-crowned teeth within the Ryazan assemblage. Further analyses of a larger number of cuspidate and un-cuspidate teeth of *Ptychodus* are thus necessary and recommended in order to better clarify the dental renewal and the durophagous tooth adaptations of this enigmatic predator.

Regardless of possible differences in dentition efficiency and functionalities between cuspidate and un-cuspidate species, the different morphologies identified in *Ptychodus* from Russia, as well as from other European localities, confirm a complex and intricate scenario for the trophic ecology of this marine predator (see also Amadori et al., 2019b, 2020b, 2022). Moreover, the various degrees of tooth wear documented for cuspidate and un-cuspidate *Ptychodus* from numerous other localities around the world indicate durophagy as one of the main feeding strategies for this extinct predatory elasmobranch, reaching even a high degree of specialization in some species (e.g., Woodward, 1887, 1912; Shimada, 2012; Diedrich, 2013; Amadori et al., 2019b, 2020a, 2022; Hamm, 2020a; present paper). In *Ptychodus*, diversification in tooth morphology and, consequently, in diet preferences could therefore have reduced competition for food (e.g., shell-covered prey) within the genus (niche specialization), as well as with other possible durophagous groups.

### Species Richness across Europe: A Preliminary Comparison

*Ptychodus* is well known from the Upper Cretaceous of Europe with numerous isolated and associated teeth coming from the Cenomanian-Campanian of various localities, such as England, France, Belgium, Germany, Poland, and Italy (e.g., Agassiz, 1835; Leriche, 1902, 1906; Woodward, 1912; Herman, 1977; Niedźwiedzki and Kalina, 2003; Diedrich, 2013; Amadori et al., 2019a, 2019b, 2020a, b; see Table S3 in Supplemental Data, for the updated record of *Ptychodus* from Europe). The most abundant assemblages of *Ptychodus* are reported from England and France (e.g., Leriche, 1902, 1906; Woodward, 1912; Herman, 1977). *Ptychodus* from England is rather species diverse with nine species reported from the Cenomanian–Campanian, but the occurrences are mainly limited to the southern area of England (see Table 1). Northern France shows a higher diversity than the southern regions of France, with nine species of *Ptychodus* being identified in the Cenomanian–Campanian (see Table 1). An isolated tooth (coll. Déchaux) of *P*. *decurrens* from the Albian of Clansayes (Auvergne-Rhône-Alpes region, southern France) and housed in the fossil collection at the “Faculté des Sciences de Grenoble” (Université Grenoble Alpes, France; see Priem, 1912: 255) represents the oldest unambiguous occurrence of the genus that has been documented from Europe up to now. In addition, other isolated tooth fragments from the Gault Formation (Albian) of Cucheron (Chartreuse area, Auvergne-Rhône-Alpes region, southeastern France) and belonging to the Collections géologiques de l’Observatoire des Sciences de l’Univers de Grenoble are claerly assignable to *Ptychodus* (see also Breistroffer, 1933). *Ptychodus* also was reported from the Albian of Rencurel in the Auvergne-Rhône-Alpes region of southern France (Priem, 1912) and the Albian–Turonian (Bristow, 1998) of the Mondrepuis area in northern France (Leriche, 1906; Herman, 1977). The cited material, however, was not figured and it therefore is not possible to correctly identify the specimens. These occurrences are therefore discarded from the present study until a future and desirable revision of the French records of *Ptychodus* has been conducted.

Although the fossil record of *Ptychodus* from northern Italy and Belgium is relatively rare compared with that from England and France, 8–9 taxa of *Ptychodus* previously were identified from the Cenomanian–Santonian (possibly Campanian) of these areas (see Table 1). *Ptychodus* remains are well documented from both western and eastern German localities with abundant, but less diverse, assemblages from the Cenomanian to Campanian; in particular, cuspidate taxa are quite rare (see Table 1). A relatively high number of species of *Ptychodus* (5–6) have been documented from the Cenomanian–Turonian of northern Czech Republic and southern Poland, respectively, up to now (see Table 1), based on very rare isolated teeth.

Based on previous records and the material documented in the present study, six species of *Ptychodus* certainty occurred in the Cenomanian–?Santonian of European Russia (see also Table 2). Additionally, ?*P*. *marginalis* and ?*P*. *mediterraneus* are documented only from the Ryazan and Saratov regions. Un-cuspidate taxa, such as *P*. *decurrens*, *P*. *latissimus*, and *P*. *polygyrus*, are the most widespread in western European Russia together with the cuspidate *P*. *mammillaris*. The rarest species of *Ptychodus* from the Upper Cretaceous of Russia are *P*. *altior* and ? *P*. *marginalis* and their distribution seems to be limited to the Ryazan Oblast area (see also Table 2). New selachian dental remains, including an isolated tooth of *P*. *rugosus*, from the Upper Cretaceous (probably Santonian–Campanian) of the Orenburg region (eastern European Russia) is currently under reassessment (Jambura et al., in prep.). This confirms the presence of *P*. *rugosus* in European Russia, which previously had been reported from the Saratov area (see Sinzow, 1899).

**Figure 11:**
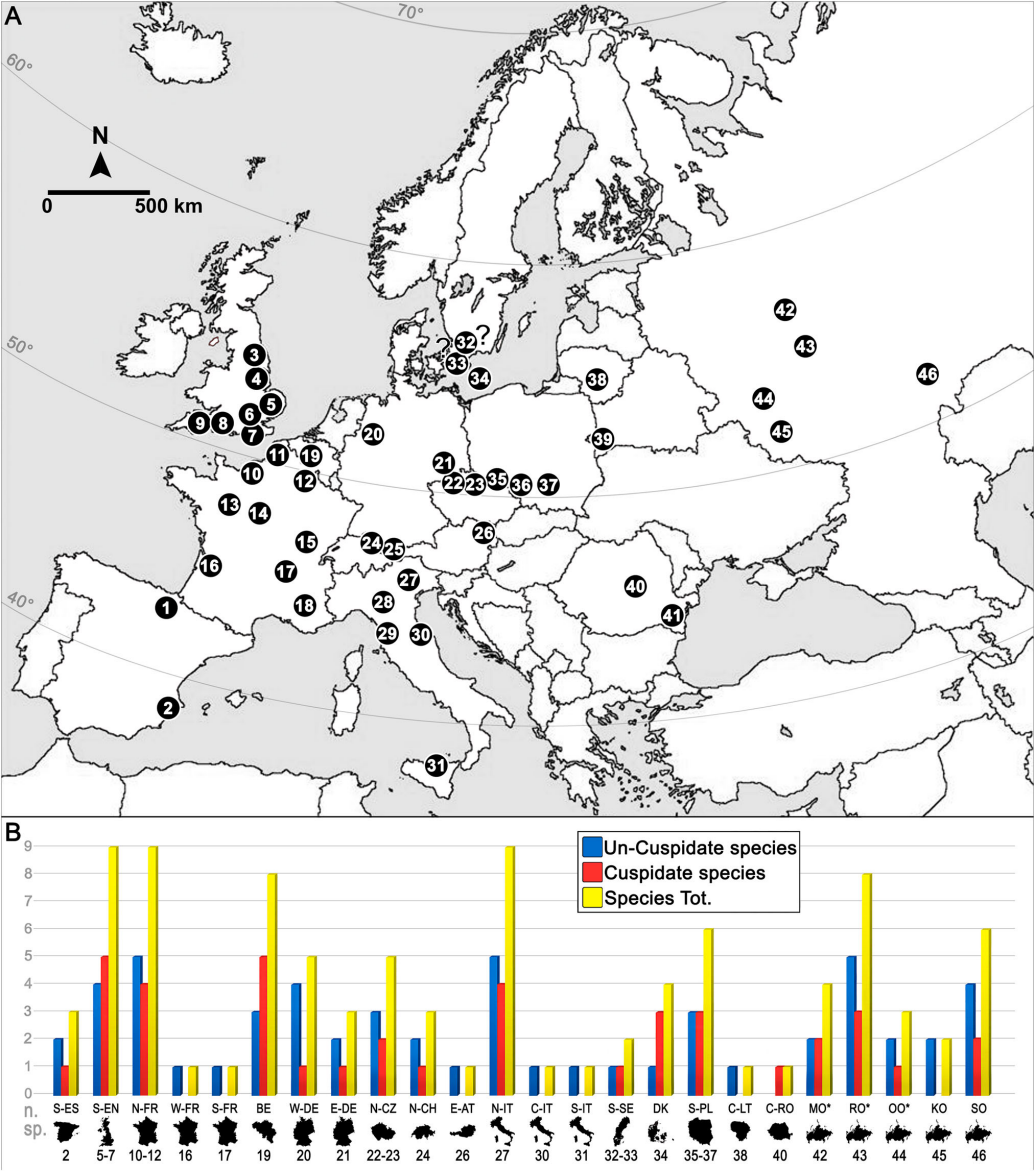
European localities that yielded teeth of *Ptychodus* (**A**) with a comparison between the species richness (**B**) between main fossiliferous areas (**BE**, Belgium; **C-IT**, central Italy; **C-LT**, central Lithuania; **C-RO**, **DK**, Denmark; central Romania; **E-AT**, eastern Austria; **E-DE**, eastern Germany; **KO**, Kursk Oblast, Russia; **MO**, Moscow Oblast; **N-FR**, northern France; **N-CH**, northern Switzerland; **N-CZ**, northern Czech Republic; **N-IT**, northern Italy; **OO**, Oryol Oblast; **RO**, Ryazan Oblast; **S-ES**, southern Spain; **S-EN**, southern England; **S-FR**, southeastern France; **S-IT**, southern Italy; **S-PL**, southern Poland; **S-SE**, southern Sweden; **SO**, Saratov Oblast; **W-DE**, western Germany; **W-FR**, western France; see also Tables 1 and 2, and Tables S3 and Table S4 in Supplemental Data. Asterisks indicate the areas from which the material examined in this study comes. **1**, western Pyrenees (Spain); **2**, Ferriol-Elche (Spain); **3**, Yorkshire (England); **4**, Lincolnshire (England); **5**, Suffolk, Norfolk, Essex (England); **6**, Bedfordshire, Hertfordshire, Buckinghamshire (England); **7**, Sussex, Surrey, Kent, Greater London (England); **8**, Isle of Wight, Hampshire, Wiltshire, Dorset (England); **9**, Devon (England); **10**, Normandy (France); **11**, Hauts-de-France (France); **12**, Grand Est (France); **13**, Pays de la Loire (France); **14**, Centre-Val de Loire (France); **15**, Bourgogne-Franche-Comté (France); **16**, New Aquitaine (France); **17**, Auvergne-Rhône-Alpes (France); **18**, Provence-Alpes-Côte d’Azur (France); **19**, Hainaut, Namur (Belgium); **20**, Westphalia (Germany); **21**, Saxony (Germany); **22**, Úpohlavy (Czech Republic); **23**, Benátky nad Jizerou, Březina, Lysá nad Labem (Czech Republic); **24**, Oberriet, St. Gallen (Switzerland); **25**, Vorarlberg (Austria); **26**, Vienna (Austria); **27**, Veneto (Italy); **28**, Emilia-Romagna (Italy); **29**, Tuscany (Italy); **30**, Marche (Italy); **31**, Sicily (Italy); **32**, Oretorp (Sweden); **33**, Annetorp (Sweden); **34**, Bornholm Island (Denmark); **35**, Wyszki (Poland); **36**, Opole, Włodzienin (Poland); **37**, Glanów, Mydlniki, Sobkow (Poland); **38**, Kaunas, Skirsnemunė (Lithuania); **39**, Brèst (Belarus); **40**, Ormeniş (Romania); **41**, Peştera (Romania); **42**, Moscow Oblast (Russia); **43**, Ryazan Oblast (Russia); **44**, Oryol Oblast (Russia); **45**, Kursk Oblast (Russia); **46**, Saratov Oblast (Russia).

A maximum of 10 species of *Ptychodus (P. altior, P. anonymus, P. decurrens, P. latissimus, P. mammillaris, P. marginalis, P. mediterraneus*, *P*. *mortoni*, *P*. *polygyrus*, and *P*. *rugosus*) are currently known from Europe and reported in 15 countries (see Tables 1 and 2), with *P*. *decurrens*, *P*. *latissimus*, and *P*. *mammillaris* being the most widespread taxa (see also Table S3 in Supplemental Data). *Ptychodus mortoni* is instead very rare with a few isolated teeth only occurring in England, Belgium, and Italy (see also Table 1). Moreover, *P*. *mortoni* has not been identified from any Russian locality so far (see also Table 2). *Ptychodus mediterraneus* only has been reported from northern France and northern Italy up to now. This species, however, possibly reached European Russia as well (see also Tables 1 and 2). Due to the dubious provenance of the *Ptychodus* specimens reported from southern Sweden (see also Siverson, 1993), the teeth described here from the Cenomanian of the Varavinsky Ravine locality (Moscow Oblast, European Russia) seem to represent the northernmost occurrences documented in Europe for this durophagous predator in the early Late Cretaceous so far (see Fig. 11A). The diversity hot spots for *Ptychodus* in Europe are southern England, northern France, and northern Italy (see Fig. 11B). In addition, the *Ptychodus* assemblage from the Upper Cretaceous of the Ryazan Oblast described in the present study seems to be the most diverse among those from the Upper Cretaceous of European Russia (see Fig. 11B). Both cuspidate and un-cuspidate species were well distributed across all the examined European areas, with a few exceptions (see Fig. 11B). Among the Russian localities, no cuspidate species has been reported from the Kursk Oblast (European Russia) so far (see Fig. 11B; see also Table 2).

### Paleoenvironment and Paleobiogeography

Scenarios for modern marine ecosystems based on models of climate and environmental change and community responses are increasingly alarming (Bruno et al., 2018; Ullah et al., 2018; Babcock et al., 2019). Drastic fluctuations in environmental conditions, as well as loss and degradation of suitable habitats, significantly affect the distribution patterns and dispersal performances of extant elasmobranchs (Musick et al., 2000; De Angelis et al., 2008; Field, 2009; Knip et al., 2010). For instance, the water temperature and salinity represent crucial environmental factors that drive distributions and habitat preferences of extant elasmobranchs, also directly influencing their physiology (e.g., muscle performance and metabolism; Bernal et al., 2012; Schlaff et al., 2014; Munroe et al., 2014; Bernal and Lowe, 2015; Meese and Lowe, 2019). Furthermore, multiple biotic variables, such as prey density and availability, can trigger distribution changes and habitat choice for both specialized (e.g., durophagous batoids) and generalist elasmobranchs (Torres et al., 2006; Pimiento et al., 2016; Serrano-Flores et al., 2019). In particular, variations in biotic and abiotic factors severely threaten the survival of specialized taxa, and identifying specialized predators is thus crucial to properly evaluate the vulnerability of elasmobranch communities (Munroe et al., 2014; Pimiento et al., 2016). However, abiotic stressors may alter the abundance and distribution of their primary prey, indirectly influencing dispersal patterns (Schlaff et al., 2014). Such strong connections between elasmobranchs, including some durophagous taxa, and their environment can be assumed throughout most of their evolutionary history (e.g., Kriwet and Klug, 2008; Sorenson et al., 2014; Maduna et al., 2020). Valuable information on paleogeographic and climatic stressors driving the distribution and evolution of a fauna over long temporal scales can be inferred from its paleobiogeographic trends (Brooks and McLennan, 1991; Brown and Lomolino, 1998; Lieberman, 2000; Myers and Lieberman, 2010; Maduna et al., 2020). Studies on spatial and temporal distribution patterns based on fossil occurrences thus play a key role in understanding evolutionary processes, as well as in estimating conservation potential, for land and marine fauna communities (e.g., Lieberman, 2008, 2012; Louys, 2012; Peterson and Lieberman, 2012; Cranbrook and Piper, 2013; Dombrosky, 2015; Maduna et al., 2020).

As a result of the Cenomanian transgression, an epicontinental sea covered a vast area of the Eastern European Platform (Russian Platform), though for the most part it was shallow with rapidly changing currents. In the south, there were large islands that weathered and contributed sediment and debris to the seabed. During the Cenomanian, the Russian sea was widely connected in the south to the Caucasus, in the east to the Kopet Dagh seas, and in the west by seaways to Central Europe (Vinogradov, 1975). The upper Cenomanian sediments on the Russian Platform were partially eroded during the latest Cenomanian–early Turonian regression. During the early Turonian, erosion resulted in part from a sea-level fall in most regions of the Russian, Scythian, and Turonian platforms. Only in the southernmost areas did the sedimentation continue. The next transgressive episode began in the middle Turonian with a maximum in the late Turonian and Coniacian (Baraboshkin et al., 2003). Although there were episodes of regression during the Coniacian, the marine basin on the Russian Platform was somewhat reduced, especially on the outskirts of synclines and anticlines (Vinogradov, 1975). In the late Coniacian to Santonian, a vast transgression of the sea from the east and southeast covered almost completely the central Russian Platform. During the Santonian, the eastern edge of the platform was most intensively submerged and here, a narrow strait formed that connected the Russian with the boreal seas (Kuzmin et al., 2015). In the Eastern European Platform, all Late Cretaceous units with marine sediments were deposited in the northeastern peri-Tethyan basin, which was well connected to the West Siberian Sea and to the marine basins of Central Europe (Baraboshkin et al., 2003).

The early Campanian featured the largest transgression on the Russian Platform during the Upper Cretaceous (Baraboshkin et al., 2003). However, since the middle or late Campanian, the Russian Sea decreased in size and finally the central part of the Russian platform fell dry. This is clearly observable in the area of the Moscow syncline and in the east of the platform, where the direct connection to the boreal and south seas probably ceased. However, in other parts of the platform, the situation continued to be the same as before (Vinogradov, 1975).

The late Maastrichtian featured a regressive phase on the Russian Platform. The northern coastline moved south over several hundred kilometres, and sediments on coastal parts of the basin were later eroded (Baraboshkin et al., 2003). In general, frequent shallowing of the marine basin and depositional hiatuses occurred during the Upper Cretaceous on the Russian platform as a result of fluctuations in sea levels evidenced by the presence of the phosphorite content of Upper Cretaceous sediments (Sidorenko et al, 1971; Vinogradov, 1975; Baraboshkin et al., 2003; Kuzmin et al., 2015). In the center of the eastern European Platform, in a shallow-water environment with a zone of increased water turbulence, sandstones accumulated, which were saturated with inclusions of grus, sandstone gravel, and phosphorite pebbles rich in fossils including skeletal elements and teeth of marine reptiles. In addition to the remains of herpetofauna in the formations of that age, the vertebrae and teeth of cartilaginous fishes, mainly elasmobranchs are present (Sidorenko et al., 1971; Glikman, 1980; Arkhangelsky et al., 2008; Kuzmin et al., 2015).

**Figure 12:**
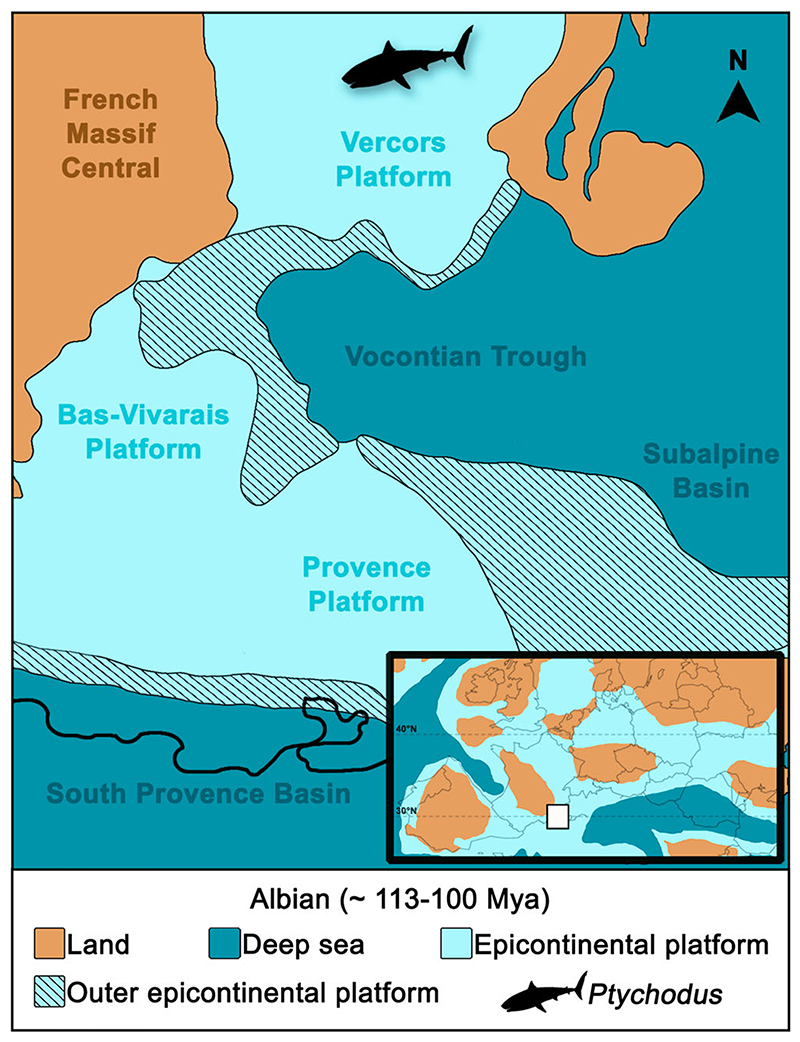
Lower Cretaceous paleogeographic maps with occurrences of *Ptychodus* from the Albian of Clansayes (Auvergne-Rhône-Alpes region, southern France; modified from Granier, 2017; see also Table S3 and Table S4 in Supplemental Data).

**Figure 13:**
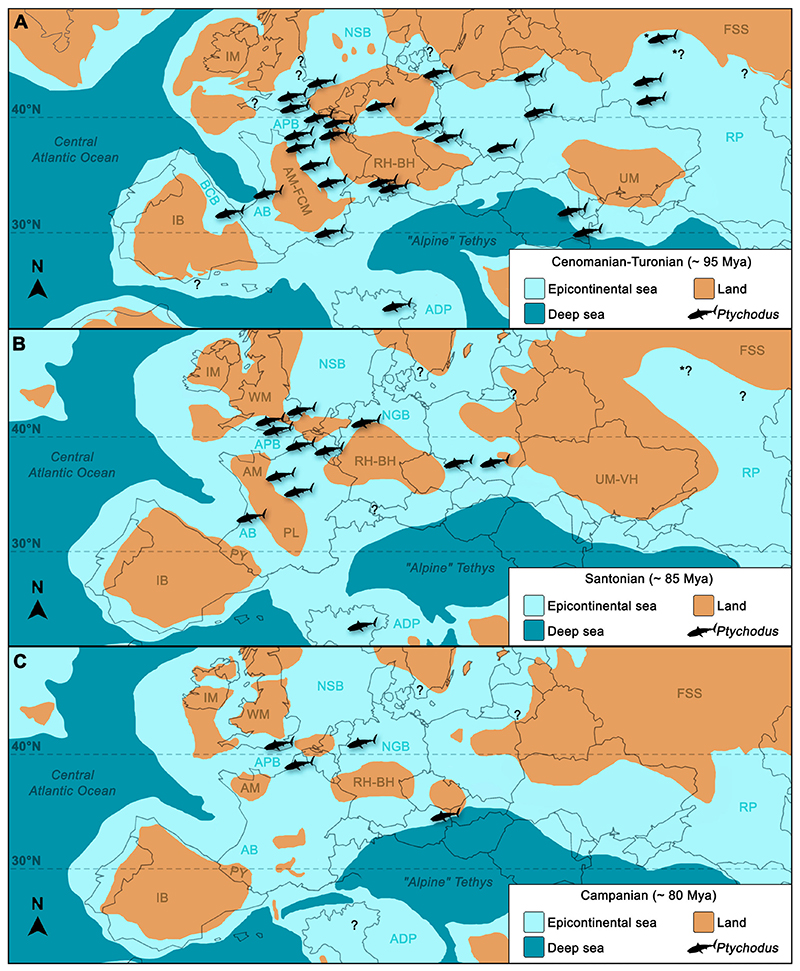
Upper Cretaceous paleogeographic maps (coastlines based on Neubauer and Harzhauser, 2022; modified from Puértolas-Pascual et al., 2016) with Cenomanian–Turonian (**A**), Santonian (**B**), and Campanian (**C**) occurrences of *Ptychodus* in Europe (see also Table S3 and Table S4 in Supplemental Data). Asterisks indicate the areas from which the material examined in this study comes. Question marks indicate occurrences with uncertain age. **Abbreviations**: **AB**, Aquitaine Basin; **ADP,** Adria Platforms; **APB**, Anglo-Paris Basin; **AM**, Armorican Massif; **BCB**, Basque-Cantabrian Basin; **IB**, Iberia; **IM**, Irish Massif; **FCM**, French Massif Central; **FSS**, Fenno-Scandian Shield; **NGB**, North German Basins; **NSB**, North Sea Basin; **PL**, Provencal Landmass; **PY**, Pyrenean Landmass; **RH-BH**, Rhenish-Bohemian High; **RP,** Russian Platform; **UM-VH**, Ukrainian Massif-Voronezh High; **WM**, Welsh Massif.

In the Cretaceous, *Ptychodus* spanned most of the peri-Tethys with a stratigraphic distribution ranging from the Albian–Cenomanian to the Campanian and thus survived for around 30–40 million years (see also ‘Spatial and temporal distribution of *Ptychodus* from Europe’ section, above). The oldest European specimens of *Ptychodus* from the Albian of Auvergne-Rhône-Alpes region (southeastern France) indicate that the rise of the peri-Tethyan domain of this durophagous predator would have started in shallow open marine environments (see also Herrle et al., 2003; Granier et al., 2017). In particular, the Early Cretaceous spatial distribution of *Ptychodus* included the epicontinental sea area of Vercors platforms (see Fig. 12). The central position of the epicontinental seas in southern French and their multiple connections with the rest of the marginal areas of the western Neo-Tethys Ocean during the Late Cretaceous would have greatly favored a subsequent spread of *Ptychodus* species throughout the entire peri-Tethys (see Fig. 13). During the Cenomanian–Turonian, *Ptychodus* was well diversified with both cuspidate and un-cuspidate species being widely distributed throughout European epicontinental seas (see Fig. 13A). In particular, this durophagous elasmobranch reached the northeastern area of the Russian platform area (RP in Fig. 13A) in the early Late Cretaceous, probably using this epicontinental seaway to migrate along the peripheral areas of the Neo-Tethys Ocean (e.g., Asian peri-Tethys) and to access the northwestern margin of the paleo-Pacific Ocean. *Ptychodus* was reported from the Turonian–Santonian of western Kazakhstan by Glickman et al. (1970) and Zhelezko and Glikman (1971; see also Popov, 2016), and the Cenomanian–Turonian to ?Campanian of Japan by Goto et al. (1996). In the Santonian, the Anglo-Paris basin, the Adria platform, and the southern Central Polish basin certainly were part of the epicontinental areas inhabited by *Ptychodus* (see Fig. 13B). The evolutionary history of *Ptychodus* in Europe extends into the Campanian of the Anglo-Paris (APB in Fig. 13C) and western North German basins (NGB in Fig. 13C). Other Campanian epicontinental seas occupied by this predatory elasmobranch include areas of present-day eastern Austria and, possibly, southern Lithuania. Additional studies of specimens of *Ptychodus* from the Upper Cretaceous of European Russia nevertheless are mandatory to establish the persistence of this durophagous elasmobranch in the Russian platform area after the Cenomanian. A serious reduction in the geographic distribution of *Ptychodus* in the peri-Tethyan seas undeniably occurred towards the end of the Upper Cretaceous.

Jouve et al. (2017:fig. 4) reconstructed a global decrease in sea surface temperature alternating with temporary water heating during the Late Cretaceous. The highest heating peak in the shallow seas occurred in the Turonian, whereas the late Late Cretaceous saw a dramatic cooling that ended at the end of the Mesozoic era; although the temperature decline of surface waters already started in the late Turonian, a temporary, but noticeable, warming of the shallow seas occurred in the Coniacian-upper Santonian (Jouve et al., 2017). Furthermore, a local cooling and a salinity drop in surface waters (0–1000 m depth) recently was hypothesized throughout the Upper Cretaceous in most peri-Tethyan seas at a latitude ranging between 30–60°N (see Ladant et al., 2020:fig. 10d). However, the surface water temperature was relatively stable in the westernmost peri-Tethys, also rising in some areas (e.g., North German Basin; see Ladant et al., 2020:fig. 9a). The heating of these shallow water areas probably were due to the superficial ‘proto-Gulf stream’, which distributed warm surface waters from the Gulf of Mexico towards northwestern Europe during the Late Cretaceous (see Ladant et al., 2020; Wilmsen et al., 2021).

Variations in temperature and salinity represent typical abiotic stressors affecting the geographic distribution and migration patterns of extant elasmobranchs including durophagous taxa, such as *Myliobatis* (eagle rays) and *Mustelus* (smooth-hound sharks; Bernal et al., 2012; Schlaff et al., 2014; Bernal and Lowe, 2015; Meese and Lowe, 2019). The cooling of the epicontinental shallow seas inhabited by *Ptychodus* (see above) was probably involved in the narrowing of the European geographic range of this durophagous elasmobranch throughout the Late Cretaceous.

Conversely, the warmer waters of the ‘proto-Gulf stream’, which accessed the peri-Tethyan seas through the northern Anglo-Paris and the Aquitaine basins (see also Wilmsen et al., 2021:fig. 10), allowed *Ptychodus* species to persist in the northwestern peri-Tethys until the Campanian. However, the cooling of the sea surface alone can hardly explain the demise of *Ptychodus* in Europe after the Campanian. For example, the Late Cretaceous distribution and/or abundance of possible shelled prey (biotic factors), such as ammonites, bivalves, and crustaceans, may have been an additional factor playing a key role in the disappearance of this durophagous predator. Therefore, future surveys on possible correlations between paleobiogeography and diversity/abundance fluctuations of *Ptychodus* species and its prey in the peri-Tethys are required for confirming or discarding the hypothesis proposed in the present study.

## Conclusions

The new Late Cretaceous assemblages of *Ptychodus* from European Russia presented herein indicate the occurrence of at least seven species, *P*. *altior*, *P*. *anonymus*, *P*. *decurrens*, *P*. *latissimus*, *P*. *mammillaris*, *P*. *marginalis*, and *P*. *polygyrus* within the ?Cenomanian–Santonian shallow water environments of the Russian platform (northeastern peri-Tethys). The occurrence of an eighth additional taxon (*P*. *mediterraneus*) is likely, but requires further investigation. Two of the species here documented, *P*. *altior* and *P*. *anonymus* are reported here for the first time from the Russian localities. *Ptychodus* appeared in Europe in the late Early Cretaceous, rapidly colonizing most of the epicontinental seas of the western peri-Tethys throughout the Late Cretaceous. In the Late Cretaceous, *Ptychodus* started to migrate towards the easternmost peripheral areas of the Neo-Tethyan Ocean across the Russian platform. The highly diverse fauna of *Ptychodus* from the Malyy Prolom area indicates that the Russian platform provided a conducive environment (mainly shallow epicontinental seas) for the spread and diversification of this durophagous predator with various cuspidate and un-cuspidate taxa probably playing different roles in the trophic web (niche partitioning) of the northeastern peri-Tethys.

Additionally, local variations in abiotic factors, such as temperature and habitat availability driven by coastal line changes, might have trigged the narrowing of the geographic range of *Ptychodus* within relatively limited areas during the Late Cretaceous epicontinental seas (e.g., Neo-Tethyan margins). Nevertheless, this durophagous predator was probably sensitive to multiple environmental stressors. For instance, prey abundance and distribution could have been additional factors in shaping the evolution and dispersal of the most specialized taxa within the genus *Ptychodus*. Further studies on unknown fossil fish material from Russian localities might reveal an even more complex scenario for the Cretaceous diversification and dispersal of *Ptychodus*, as well as for other elasmobranch predators, in eastern peri-Tethyan seas.

## Supplementary Material

Suppl. Mat.

## Figures and Tables

**Table 1:** Taxonomic composition of *Ptychodus* from the Upper Cretaceous of various European localities. **Abbreviations**: **BE**, Belgium; **C**, cuspidate taxon; **C-IT**, central Italy; **C-LT**, central Lithuania; **C-RO**, central Romania; **DK**, Denmark; **E-AT**, eastern Austria; **E-DE**, eastern Germany; **N-CH**, northern Switzerland; **N-CZ**, northern Czech Republic; **N-FR**, northern France; **N-IT**, northern Italy; **S-EN**, southern England; **S-ES**, southern Spain; **S-FR**, southeastern France; **S-IT**, southern Italy; **S-SE**, southern Sweden; **S-PL**, southern Poland; **UC**, un-cuspidate taxon; **W-DE**, western Germany; **W-FR**, western France. See Table S3 for more details.

Species	S-ES	S-EN	N-FR	W-FR	S-FR	BE	W-DE	E-DE	N-CZ	N-CH	E-AT	N-IT	C-IT	S-IT	S-SE	DK	S-PL	C-LT	C-RO
*P*. *altior* (C)		✓	✓			✓		✓				✓				✓	?		✓
*P*. *anonymus* (C)	✓	✓	✓			✓			✓							?	✓		
*P*. *decurrens* (UC)	✓	✓	✓		✓		✓	✓	?			✓	✓	✓	✓		✓		
*P*. *latissimus* (UC)	?	✓	✓	✓		✓	✓	✓	✓			✓				✓	✓	✓	
*P*. *mammillaris* (C)		✓	✓			✓	✓		✓			✓				✓	✓		
*P*. *marginalis* (UC)		✓	✓			?	?					✓							
*P*. *mediterraneus* (UC)			✓									✓							
*P*. *mortoni* (C)		✓				✓						✓							
*P*. *polygyrus* (UC)		✓	✓			✓	✓		✓	?	✓	✓					✓		
*P*. *rugosus* (C)		✓	✓			✓						✓			✓				

**Table 2:** Taxonomic composition of *Ptychodus* from the Upper Cretaceous of five Russian localities (asterisk indicates species described in the present study). **Abbreviations**: **C**, cuspidate taxon; **UC**, un-cuspidate taxon.

Species	Kursk Oblast	Moscow Oblast	Oryol Oblast	Ryazan Oblast	Saratov Oblast
*P*. *altior* (C)				✓*	
*P*. *anonymus* (C)		✓*		✓*	
*P*. *decurrens* (UC)		✓*	✓	✓*	✓
*P*. *latissimus* (UC)	✓		✓	✓*	✓
*P*. *mammillaris* (C)		✓	✓	✓*	✓
*P*. *marginalis* (UC)				✓*	?
*P*. *mediterraneus* (UC)				?*	?
*P*. *mortoni* (C)					
*P*. *polygyrus* (UC)	✓	✓*		✓*	✓
*P*. *rugosus* (C)					?
