# Supplementary material for: The extinct shark, Ptychodus (Elasmobranchii, Ptychodontidae) in the Upper Cretaceous of central-western Russia—The road to easternmost peri-Tethyan seas
Source: J Vertebr Paleontol. Author manuscript; Available in PMC 2023 Aug 9. (PMC7614918; doi:10.1080/02724634.2022.2162909)
Supplement: Suppl. Mat. [file EMS183674-supplement-Suppl__Mat_.pdf]

Journal of Vertebrate Paleontology

The extinct shark, *Ptychodus* (Elasmobranchii, Ptychodontidae) in the Upper Cretaceous of central-western Russia – The road to easternmost peri-Tethyan seas

MANUEL AMADORI,[1*] SERGEY V. SOLONIN,[2*] ALEXEY V. VODOREZOV,[2] RYAN SHELL,[3] ROBERT NIEDŹWIEDZKI,[4] and JÜRGEN KRIWET[1]

[1] University of Vienna, Department of Palaeontology, UZAII, Geozentrum, Josef-Holaubek-Platz 2, Vienna, 1090, Austria, amadorim37@univie.ac.at, juergen.kriwet@univie.ac.at;

[2]Department of Geography, Ecology and Natural Management, Ryazan State University named for S. Yesenin, Ryazan, 390000, Russia, soloninserg.inbox@mail.ru, a.vodorezov@365.rsu.edu.ru;

[3]Department of Vertebrate Paleontology, Cincinnati Museum Center, Cincinnati, 45203, USA, ryanshell501@gmail.com;

[4]Institute of Geological Sciences, University of Wrocław, Wrocław, 50-204, Poland, robert.niedzwiedzki@uwr.edu.pl

*Corresponding author

Information Classification: General

SUPPLEMENTARY SYSTEMATIC PALEONTOLOGY

Class CHONDRICHTHYES Huxley, 1880

Subclass ELASMOBRANCHII Bonaparte, 1838

Order PTYCHODONTIFORMES Hamm, 2019

Family †PTYCHODONTIDAE Jaekel, 1898

Genus †*PTYCHODUS* Agassiz, 1834

Type Species—*Ptychodus latissimus* Agassiz, 1835 (nomen protectum; see Giusberti et al., 2018).

Diagnosis—See Woodward (1912).

†*PTYCHODUS* sp. cf. †*P. ANONYMUS* Williston, 1900a

Diagnosis. See Hamm (2020:14).

Referred Material—Seventeen tooth fragments (RSU DGE 2018 RO MP-43, RSU DGE 2018 RO MP-45, RSU DGE 2019 RO MP-43, RSU DGE 2020 RO MP-22, RSU DGE 2020 RO MP-24, RSU DGE 2020 RO MP-26, RSU DGE 2020 RO MP-38, RSU DGE 2021 RO MP-15, RSU DGE 2021 RO MP-17, RSU DGE 2021 RO MP-18, RSU DGE 2021 RO MP-22, RSU DGE 2021 RO MP-26, RSU DGE 2021 RO MP-27, RSU DGE 2021 RO MP-30, KP NVF 19956/216, KP NVF 19956/217 and KP NVF 19956/218) belonging to the fossil collections of the Ryazan State University and of State Darwin Museum in Moscow.

Description—In general, tooth fragments reported in this section have dental features (e.g., general shape and ridge patterns) identical to those exhibited by the dental cusp of *Ptychodus anonymus* described in the present study (see above; see also Fig. 4). RSU DGE 2018 RO MP-43, RSU DGE 2019 RO MP-43, RSU DGE 2020 RO MP-22, RSU DGE 2021 RO MP-17, RSU DGE 2021 RO MP-22 and RSU DGE 2021 RO MP-30 have identical features to those exhibited by the cuspidate crowns of *P. anonymus* here described (e.g., RSU DGE 2020 RO MP-20 in Fig. 4B–B^{III};

Information Classification: General

see above). RSU DGE 2018 RO MP-45 and RSU DGE 2021 RO MP-26 are similar to the cusp RSU DGE 2018 RO MP-42 (Fig. 4A–A^{III}), whereas RSU DGE 2020 RO MP-38 shared the cusp shape with RSU DGE 2020 RO MP-3 (Fig. 4C–C^{I}). The cusp fragment RSU DGE 2020 RO MP-24 and RSU DGE 2020 RO MP-26 show the ridge 'loops' on the right side like those in RSU DGE 2018 RO MP-42 (see also Fig. 4A; see above). The cusp of RSU DGE 2021 RO MP-9 (Fig. 4H–H^{II}) is identical to RSU DGE 2021 RO MP-15 and RSU DGE 2021 RO MP-18. RSU DGE 2021 RO MP-27 have marginal area identical to RSU DGE 2020 RO MP-20 (see Fig. 4B–B^{II}); the cusp of RSU DGE 2021 RO MP-27 is missing. KP NVF 19956/216, KP NVF 19956/217 and KP NVF 19956/218 have shapes and ornamentations similar to those described for RSU DGE 2021 RO MP-9 (Fig. 4H–H^{II}), although they preserve only the tooth cusp. Among the cusp apexes described in this section, RSU DGE 2018 RO MP-43, RSU DGE 2018 RO MP-45, RSU DGE 2020 RO MP-24, RSU DGE 2020 RO MP-38, RSU DGE 2021 RO MP-15, RSU DGE 2021 RO MP-17, RSU DGE 2021 RO MP-18, RSU DGE 2021 RO MP-22 and KP NVF 19956/217 are slightly abraded. In particular, wear traces are on the anterior side of RSU DGE 2018 RO MP-43 and on the rounded tip of RSU DGE 2018 RO MP-45, RSU DGE 2020 RO MP-24 and RSU DGE 2020 RO MP-38. Abrasions involve the entire cusp apex in RSU DGE 2021 RO MP-15 and RSU DGE 2021 RO MP-18. Only the left side of RSU DGE 2021 RO MP-17 shows traces of wear.

Remarks—Although the fragmentary specimens described in this section display a very similar cusp to the typical one seen in teeth of *Ptychodus anonymus* (see above; see also Hamm, 2019), an unambiguous and doubtless identification is unfortunately impossible at the moment.

†*PTYCHODUS* sp. cf. †*P. POLYGYRUS* Agassiz, 1835

Diagnoses—See Hamm (2020:24).

Referred Material—Three fragmentary specimens, RSU DGE 2020 RO MP-28, RSU DGE 2021 RO MP-28 and RSU DGE 2021 RO MP-29 belonging to the fossil collection of the Ryazan State University.

Information Classification: General

Description—RSU DGE 2020 RO MP-28, RSU DGE 2021 RO MP-28 and RSU DGE 2021 RO MP-29 are fragments of tooth crowns characterized by transversal ridges with thin ends curved anteriorly. The few marginal areas visible show randomly arranged granulation.

Remarks—The few characters displayed by RSU DGE 2020 RO MP-28, RSU DGE 2021 RO MP-28 and RSU DGE 2021 RO MP-29 resemble those typical of *Ptychodus polygyrus* (see Amadori et al., 2020a; Hamm, 2020a). However, the lack of the marginal area prevents an unambiguous identification of these specimens.

†*PTYCHODUS* sp.

(Fig. S1)

Referred Material—Two worn teeth RSU DGE 2020 RO MP-16 and RSU DGE 2021 RO MP-5 and the crown fragment RSU DGE 2020 RO MP-23 belonging to the collections of the Ryazan State University.

Description—Specimen RSU DGE 2020 RO MP-16 (Fig. 11A–A^{II}) is an un-cuspidate, worn tooth with asymmetric occlusal outline. The anterior protuberance is wide and rounded, whereas the posterior sulcus is shallow. The occlusal surface of the crown is poorly preserved with the right tooth edge partially broken. No occlusal ornamentation is observable on this specimen. In posterior view (Fig. 11A^{I}), the crown is slightly raised and the root is bilobate with a shallow antero-posterior sulcus; both lateral root sides are tilted inward. In lateral view (Fig. 11A^{II}), the anterior sides of both dental crown and root are inclined forward. RSU DGE 2020 RO MP-23 is a tooth fragment exhibiting the anterior edge of a dental crown. On the side opposite the external tooth margin, two relatively thin ridges transversally cross the crown occlusal surface. RSU DGE 2021 RO MP-5 (Fig. 11B–B^{IV}) is a worn tooth exhibiting a rectangular crown with a marked and rounded anterior protuberance and a deep posterior sulcus. On the almost symmetrical crown, the occlusal ornamentations are almost completely missing due to the intense abrasion of the tooth surface. Just some transversal thin ridges are still recognizable on the center on the dental crown. In

Information Classification: General

anterior and posterior views (Fig. 11B^{I}, B^{II}), the crown exhibits a high, rounded cusp and a large antero-posterior sulcus characterizes the (bilobate) root. In lateral view (Fig. 11B^{III}), the posterior side of the crown is straight, whereas its anterior outline is tilted forward; the anterior side of the root is inclined as well. In inferior view (Fig. 11B^{IV}), the root outline follows the rectangular shape of the tooth crown.

Remarks—The asymmetrical outline of RSU DGE 2020 RO MP-16 (Fig. 11A) indicates that it belonging to a lateral tooth row of the left hemiarch. However, the correct identification of the specimen is prevented by the lack of any species-specific character recognizable (e.g., occlusal ornamentations). The specimen looks partially digested, as indicated by the worn surface and blunt and rounded dental edges. The coarse granulations randomly spread on the occlusal surface of RSU DGE 2020 RO MP-23 resemble those previously described for un-cuspidate species, such as *Ptychodus marginalis*, *P. martini* and *P. polygyrus*. Furthermore, the poorly preserved ridges do not reach the thickness usually shown by the *P. latissimus* teeth. However, the specimen is too fragmentary for any accurate identification. An original arrangement within the lower symphyseal, or most central, rows is here hypothesized for RSU DGE 2021 RO MP-5 based on its almost symmetrical crown outline and its bulged occlusal surface (see Fig. 11B). A correct identification is not possible for RSU DGE 2021 RO MP-5 due to the lack of species-specific ornamentation on the occlusal tooth surface.

Information Classification: General

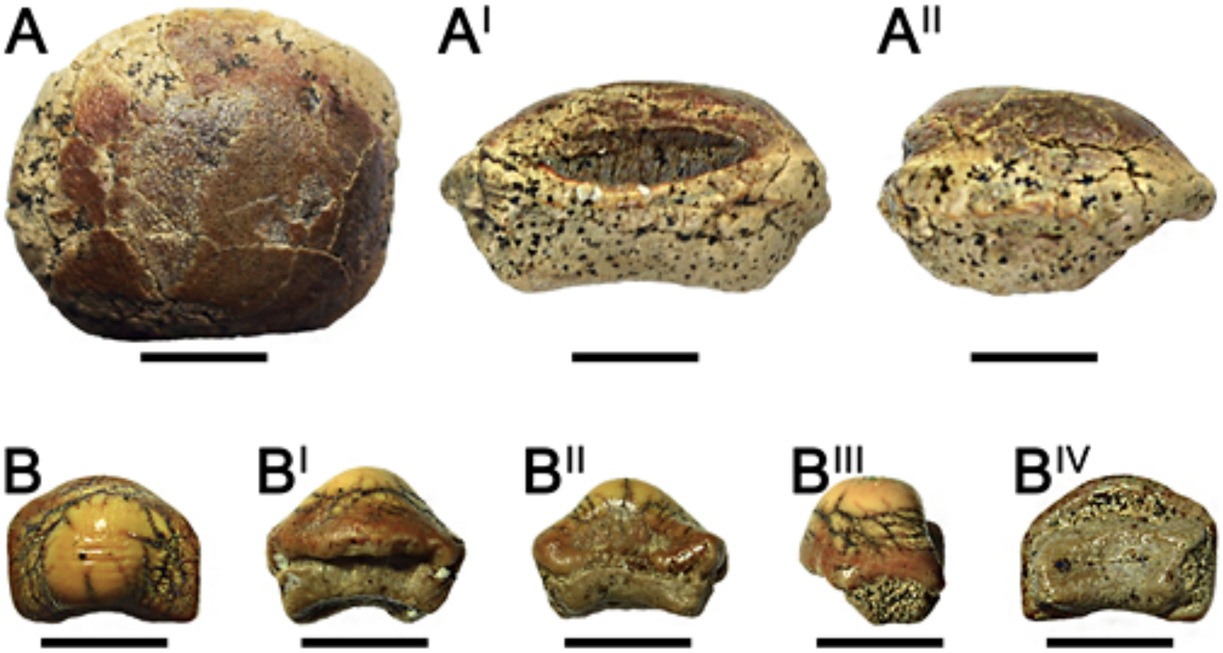

FIGURE S1. Teeth of *Ptychodus* sp. from the Upper Cretaceous of Ryazan Oblast (western Russia) in occlusal (**A**, **B**), anterior (**B^{I}**), posterior (**A^{I}**, **B^{II}**), lateral (**A^{II}**, **B^{III}**) and inferior (**B^{IV}**) views. **A–A^{II}**, RSU DGE 2020 RO MP-16; **B–B^{IV}**, RSU DGE 2021 RO MP-5. Scale bars equal 10 mm.

Information Classification: General

TABLE S1. Details and proposed identification for isolated teeth of *Ptychodus* from the Upper Cretaceous of Malyy Prolom (Ryazan Oblast, Russia). **Abbreviations**: **C**, cuspidate; **UC**, un-cuspidate; **WT**, worn tooth.

Catalogue N°	Nature of the specimen	WT	Identification
RSU DGE 2018 RO MP Ôäû-41	Tooth		*P. polygyrus* (UC)
RSU DGE 2018 RO MP Ôäû-42	Tooth	✓	*P. anonymus* (C)
RSU DGE 2018 RO MP Ôäû-43	Tooth cusp (fragment)	✓	*P.* cf. *anonymus* (C)
RSU DGE 2018 RO MP Ôäû-44	Tooth crown		*P. anonymus* (C)
RSU DGE 2018 RO MP Ôäû-45	Tooth cusp (fragment)	✓	*P.* cf. *anonymus* (C)
RSU DGE 2018 RO MP Ôäû-46	Tooth crown	✓	*P. anonymus* (C)
RSU DGE 2018 RO MP Ôäû-47	Tooth crown	✓	*P. altior* (C)
RSU DGE 2019 RO MP Ôäû-43	Tooth cusp (fragment)		*P.* cf. *anonymus* (C)
RSU DGE 2020 RO MP Ôäû-3	Tooth	✓	*P. anonymus* (C)
RSU DGE 2020 RO MP Ôäû-6	Tooth	✓	*P. mammillaris* (C)
RSU DGE 2020 RO MP Ôäû-7	Tooth crown	✓	*P. anonymus* (C)
RSU DGE 2020 RO MP Ôäû-8	Tooth crown	✓	*P. altior* (C)
RSU DGE 2020 RO MP Ôäû-9	Tooth crown	✓	*P. altior* (C)
RSU DGE 2020 RO MP Ôäû-10	Tooth		*P. decurrens* (UC)
RSU DGE 2020 RO MP Ôäû-11	Tooth		*P. decurrens* (UC)
RSU DGE 2020 RO MP Ôäû-12	Tooth crown	✓	*P. mammillaris* (C)
RSU DGE 2020 RO MP Ôäû-13	Tooth crown		*P. anonymus* (C)
RSU DGE 2020 RO MP Ôäû-14	Tooth	✓	*P. anonymus* (C)
RSU DGE 2020 RO MP Ôäû-16	Tooth (totally abraded)	✓	*Ptychodus* sp.
RSU DGE 2020 RO MP Ôäû-17	Tooth crown		*P. altior* (C)

Information Classification: General

TABLE S1. (Continued)

RSU DGE 2020 RO MP Ôäû-18	Tooth crown	✓	*P. anonymus* (C)
RSU DGE 2020 RO MP Ôäû-19	Tooth crown	✓	*P. anonymus* (C)
RSU DGE 2020 RO MP Ôäû-20	Tooth	✓	*P. anonymus* (C)
RSU DGE 2020 RO MP Ôäû-21	Tooth		*P. decurrens* (UC)
RSU DGE 2020 RO MP Ôäû-22	Tooth cusp (fragment)		*P.* cf. *anonymus* (C)
RSU DGE 2020 RO MP Ôäû-23	Tooth crown (fragment)		*Ptychodus* sp.
RSU DGE 2020 RO MP Ôäû-24	Tooth crown (fragment)	✓	*P.* cf. *anonymus* (C)
RSU DGE 2020 RO MP Ôäû-25	Tooth crown		*P. altior* (C)
RSU DGE 2020 RO MP Ôäû-26	Tooth cusp (fragment)		*P.* cf. *anonymus* (C)
RSU DGE 2020 RO MP Ôäû-27	Tooth cusp (fragment)		*P. altior* (C)
RSU DGE 2020 RO MP Ôäû-28	Tooth crown (fragment)		*P.* cf. *polygyrus* (UC)
RSU DGE 2020 RO MP Ôäû-29	Tooth (broken)	✓	*P. anonymus* (C)
RSU DGE 2020 RO MP Ôäû-38	Tooth cusp (fragment)	✓	*P.* cf. *anonymus* (C)
RSU DGE 2020 RO MP Ôäû-39	Tooth	✓	*P. altior* (C)
RSU DGE 2021 RO MP Ôäû-1	Tooth crown	✓	*P. altior* (C)
RSU DGE 2021 RO MP Ôäû-2	Tooth	✓	*P. mammillaris* (C)
RSU DGE 2021 RO MP Ôäû-3	Tooth crown	✓	*P. anonymus* (C)
RSU DGE 2021 RO MP Ôäû-4	Tooth		*P. decurrens* (UC)
RSU DGE 2021 RO MP Ôäû-5	Tooth		*Ptychodus* sp.
RSU DGE 2021 RO MP Ôäû-6	Tooth crown		*P. decurrens* (UC)
RSU DGE 2021 RO MP Ôäû-7	Tooth	✓	*P. anonymus* (C)
RSU DGE 2021 RO MP Ôäû-8	Tooth	✓	*P. anonymus* (C)
RSU DGE 2021 RO MP Ôäû-9	Tooth	✓	*P. anonymus* (C)

Information Classification: General

TABLE S1. (Continued)

RSU DGE 2021 RO MP Ôäû-10	Tooth crown	✓	*P.* cf. *mediterraneus* (UC)
RSU DGE 2021 RO MP Ôäû-11	Tooth crown		*P. decurrens* (UC)
RSU DGE 2021 RO MP Ôäû-12	Tooth crown	✓	*P. anonymus* (C)
RSU DGE 2021 RO MP Ôäû-13	Tooth		*P. decurrens* (UC)
RSU DGE 2021 RO MP Ôäû-14	Tooth crown	✓	*P. mammillaris* (C)
RSU DGE 2021 RO MP Ôäû-15	Tooth cusp (fragment)	✓	*P.* cf. *anonymus* (C)
RSU DGE 2021 RO MP Ôäû-16	Tooth crown (fragment)		*P. altior* (C)
RSU DGE 2021 RO MP Ôäû-17	Tooth cusp (fragment)	✓	*P.* cf. *anonymus* (C)
RSU DGE 2021 RO MP Ôäû-18	Tooth cusp (fragment)	✓	*P.* cf. *anonymus* (C)
RSU DGE 2021 RO MP Ôäû-19	Tooth		*P. decurrens* (UC)
RSU DGE 2021 RO MP Ôäû-20	Tooth	✓	*P. mammillaris* (C)
RSU DGE 2021 RO MP Ôäû-21	Tooth	✓	*P. anonymus* (C)
RSU DGE 2021 RO MP Ôäû-22	Tooth crown	✓	*P.* cf. *anonymus* (C)
RSU DGE 2021 RO MP Ôäû-23	Tooth crown	✓	*P. anonymus* (C)
RSU DGE 2021 RO MP Ôäû-24	Tooth crown	✓	*P. anonymus* (C)
RSU DGE 2021 RO MP Ôäû-25	Tooth	✓	*P. anonymus* (C)
RSU DGE 2021 RO MP Ôäû-26	Tooth (broken)	✓	*P.* cf. *anonymus* (C)
RSU DGE 2021 RO MP Ôäû-27	Tooth crown (fragment)		*P.* cf. *anonymus* (C)
RSU DGE 2021 RO MP Ôäû-28	Tooth crown (fragment)		*P.* cf. *polygyrus* (UC)
RSU DGE 2021 RO MP Ôäû-29	Tooth crown (fragment)	✓	*P.* cf. *polygyrus* (UC)
RSU DGE 2021 RO MP Ôäû-30	Tooth cusp (fragment)		*P.* cf. *anonymus* (C)
SS106#-1	Tooth		*P. latissimus* (UC)
SS106#-2	Tooth		*Ptychodus* sp.
SS106#-3	Tooth		*P.* cf. *marginalis* (UC)

Information Classification: General

TABLE S1. (Continued)

SS106#-4	Tooth	✓	*P. mammillaris* (C)
SS106#-7	Tooth crown		*P. mammillaris* (C)

Information Classification: General

TABLE S2. Details and proposed identification for isolated teeth of *Ptychodus* from the Upper Cretaceous of Varavinsky ravine (Moscow Oblast, Russia). **Abbreviations**: **C**, cuspidate; **UC**, un-cuspidate; **WT**, worn tooth.

Catalogue N°	Nature of the specimen	WT	Identification
KP NVF 19956/16	Broken Tooth crown	✓	*P. decurrens* (UC)
KP NVF 19956/214	Broken Tooth crown	✓	*P. anonymus* (C)
KP NVF 19956/215	Broken Tooth crown	✓	*P.* cf. *polygyrus* (UC)
KP NVF 19956/216	Broken Tooth crown		*P.* cf. *anonymus* (C)
KP NVF 19956/217	Tooth cusp (fragment)	✓	*P.* cf. *anonymus* (C)
KP NVF 19956/218	Broken Tooth crown		*P.* cf. *anonymus* (C)

Information Classification: General

TABLE S3. Summary of the European record of *Ptychodus* with original (OTI) and reviewed (RTI) taxonomical identification. **Abbreviations**: **Alb**, Albian; **Cam**, Campanian; **Cen**, Cenomanian; **Con**, Coniacian; **Ref**, references;; **San**, Santonian; **Tur**, Turonian; **UCr**, Upper Cretaceous.

OTI	RTI	Age (Ref)	Locality	Country	Ref
P. granulosus	*P. polygyrus*	Cam	Vienna area	Austria (eastern)	49
?*P. polygyrus*	?*P. polygyrus*	Tur	Vorarlberg area	Austria (western)	49
?*P. mammillaris*	?*P. mammillaris*	Tur	Brest area	Belarus (southern)	39
?*Ptychodus* sp.	?*Ptychodus* sp.	Cen	Brest area	Belarus (southern)	39
P. elevatus	*P. altior*	Con	Hainaut province	Belgium (western)	25
P. mammillaris var. *anonymus*	*P. anonymus*	Tur	Hainaut province	Belgium (western)	25
P. latissimus	*P. latissimus*	Con-San	Namur province	Belgium (western)	25
?*P. decurrens*	?*P. decurrens*	Cen-Tur	Hainaut province	Belgium (western)	25
P. mammillaris	*P. mammillaris*	Cen	Hainaut province	Belgium (western)	30
P. mammillaris var. *mammillaris*	*P. mammillaris*	Tur	Hainaut province	Belgium (western)	25
P. elevatus	*P. cf. P. marginalis*	Con	Hainaut province	Belgium (western)	25
P. mortoni	*P. mortoni*	Con-San	Namur province	Belgium (western)	25
P. polygyrus	*P. polygyrus*	Con-San	Namur province	Belgium (western)	25
P. paucisulcatus	*P. polygyrus*	Con	Hainaut province	Belgium (western)	25
P. rugosus	*P. rugosus*	Con-San	Namur province	Belgium (western)	25
?*P. rugosus*	?*P. rugosus*	San	Hainaut province	Belgium (western)	25
P. mammillaris	*P.* cf. *P. anonymus*	Tur	Úpohlavy area	Czech Republic (northern)	54
P. anonymus	*P. anonymus*	Cen	Březina area	Czech Republic (northern)	51
P. cf. *P. decurrens*	*P.* cf. *P. decurrens*	Cen	Březina area	Czech Republic (northern)	51

Information Classification: General

TABLE S3. (Continued)

P. latissimus	*P. latissimus*	Tur	Úpohlavy area	Czech Republic (northern)	54
P. latissimus	*P. latissimus*	Tur	Benátky nad Jizerou	Czech Republic (northern)	8, 50
P. mammillaris	*P. mammillaris*	Tur	Lysá nad Labem	Czech Republic (northern)	8, 50
P. polygyrus	*P. polygyrus*	Cen	Březina area	Czech Republic (northern)	51
P. cf. *P. occidentalis*	*P.* cf. *P. decurrens*	Cen	Březina area	Czech Republic (northern)	51
P. cf. *P. latissimus*	*P. polygyrus*	Cen	Březina area	Czech Republic (northern)	51
P. altior	*P. altior*	Con	Bornholm Island	Denmark	21
P. cf. *P. rugosus*	*P.* cf. *P. anonymus*	Alb-Cen (22)	Bornholm Island	Denmark	44
P. latissimus	*P. latissimus*	Con	Bornholm Island	Denmark	21
P. mammillaris	*P. mammillaris*	Con	Bornholm Island	Denmark	21
P. altior	*P. altior*	"Sen"	Sussex	England (southern)	55
P. mammillaris	*P. anonymus*	Cen (18)	Kent	England (southern)	32
?*P. anonymus*	?*P. anonymus*	Tur-Con	Kent	England (southern)	25
P. decurrens	*P. decurrens*	Cen-Tur (18)	Kent, Sussex	England (southern)	55
P. latissimus	*P. latissimus*	Tur (18)	Hertford, Kent, Suffolk, Sussex	England (southern)	53, 55
?*P. latissimus*	?*P. latissimus*	Cen-San	Buckinghamshire, Hampshire, Hertfordshire, Kent, Norfolk, Suffolk, Surrey, Sussex, Wiltshire	England (southern)	32
?*P. mammillaris*	?*P. mammillaris*	Cen-Con (18)	Bedfordshire, Devon, Dorset, Hertfordshire, Wight Island, Kent, Lincolnshire, Sussex, Surrey, Wiltshire, Yorkshire	England (central and southern)	25, 32
P. mammillaris	*P. mammillaris*	Tur	Sussex	England (southern)	55

Information Classification: General

TABLE S3. (Continued)

P. concentricus	*P. marginalis*	Cen	Kent	England (southern)	32
P. polygyrus var. *marginalis*	*P. marginalis*	Tur (18)	Kent, Sussex	England (southern)	55
?*P. marginalis*	?*P. marginalis*	Tur-Con	Kent	England (southern)	32
P. mortoni	*P. mortoni*	UCr	Sussex	England (southern)	16
P. polygyrus	*P. polygyrus*	Con-San	Kent, Surrey	England (southern)	8, 55, 56
?*P. polygyrus*	?*P. polygyrus*	Con-Cam	Essex, Greater London, Hampshire, Kent, Surrey, Wiltshire	England (southern)	32
P. rugosus	*P. rugosus*	Con-San (18)	Kent, Surrey	England (southern)	55
P. rugosus	*P. altior*	Tur-Con	Hauts-de-France	France (northern)	30, 40
P. rugosus	*P. altior*	Tur-Con	Grand Est	France (northern)	23
P. mammillaris	*P.* cf. *P. altior*	Cen	Normandy	France (northern)	42
P. anonymus	*P. anonymus*	Cen-Tur	Normandy	France (northern)	8, 38
P. mammillaris	*P. anonymus*	Tur	Grand Est	France (northern)	23
P. rugosus	*P. anonymus*	Cen	Le Havre area	France (northern)	26
P. concentricus	*P.* cf. *P. anonymus*	Cen (18)	Hauts-de-France	France (northern)	25
P. mammillaris var. *anonymus*	*P. anonymus*	Cen (18)	Hauts-de-France	France (northern)	25
Ptychodus sp.	*P. decurrens*	Cen	Normandy	France (northern)	26
P. decurrens var. *decurrens*	*P. decurrens*	Cen	Hauts-de-France	France (northern)	25
P. decurrens var. *multiplicatus*	*P. decurrens*	Tur	Hauts-de-France	France (northern)	25
P. decurrens	*P. decurrens*	Cen	Hauts-de-France	France (northern)	30
?*P. decurrens*	?*P. decurrens*	Cen	Bourgogne-Franche-Comté	France (central)	42
?*P. decurrens*	?*P. decurrens*	Cen	Provence-Alpes-Côte d'Azur	France (southeastern)	42
?*P. decurrens*	?*P. decurrens*	Con-San	Pays de la Loire	France (central)	42
P. multistriatus	*P. decurrens*	Cen	Normandy	France (northern)	30

Information Classification: General

TABLE S3. (Continued)

?*P. decurrens*	?*P. decurrens*	Alb-Tur (9)	Hauts-de-France	France (northern)	25, 30
P. decurrens	*P. decurrens*	Alb	Auvergne-Rhône-Alpes	France (southeastern)	20, 43
P. cf. *P. latissimus*	*P. latissimus*	Cen-Tur	Normandy	France (northern)	8, 38
P. latissimus	*P. latissimus*	Tur	New Aquitaine	France (western)	53
P. latissimus	*P. latissimus*	Tur	Hauts-de-France	France (northern)	41
P. latissimus	*P. latissimus*	Cen (18)	Hauts-de-France	France (northern)	25
P. latissimus	*P. latissimus*	Tur	Hauts-de-France	France (northern)	30
?*P. latissimus*	?*P. latissimus*	Con-Cam (47, 11, 25)	Hauts-de-France	France (northern)	30
?*P. paucisulcatus*	?*P. latissimus*	Con-San	Hauts-de-France	France (northern)	25
P. mammillaris var. *mammillaris*	*P. mammillaris*	Cen (18)	Hauts-de-France	France (northern)	25
P. mammillaris	*P. mammillaris*	Tur	Grand Est	France (northern)	23
P. mammillaris	*P. mammillaris*	Cen	Normandy	France (northern)	42
?*P. mammillaris*	?*P. mammillaris*	Tur	Bourgogne-Franche-Comté	France (central)	42
?*P. mammillaris*	?*P. mammillaris*	Tur	Pays de la Loire	France (central)	42
?*P. mammillaris*	?*P. mammillaris*	Con (25)	Hauts-de-France	France (northern)	30
?*P. mammillaris*	?*P. mammillaris*	Con-San	Normandy	France (northern)	42
P. marginalis	*P. marginalis*	Cen (18)	Hauts-de-France	France (northern)	25
P. concentricus	*P. marginalis*	Cen (18)	Hauts-de-France	France (northern)	25, 30, 31
?*P. marginalis*	?*P. marginalis*	Tur	Hauts-de-France	France (northern)	25
P. polygyrus	*P. mediterraneus*	Con	Hauts-de-France	France (northern)	30
P. polygyrus var. *marginalis*	*P. mediterraneus*	Cam (47, 11)	Hauts-de-France	France (northern)	30
?*P. belluccii*	?*P. polygyrus*	Cam (47, 11)	Hauts-de-France	France (northern)	25
P. latissimus	*P. polygyrus*	Tur	Hauts-de-France	France (northern)	25
P. polygyrus	*P. polygyrus*	Con	Hauts-de-France	France (northern)	30

Information Classification: General

TABLE S3. (Continued)

?*P. polygyrus*	?*P. polygyrus*	?Alb	Auvergne-Rhône-Alpes	France (southeastern)	43
?*P. polygyrus*	?*P. polygyrus*	Cen	Pays de la Loire	France (central)	42
?*P. polygyrus*	?*P. polygyrus*	Con-San	Bourgogne-Franche-Comté	France (central)	42
?*P. polygyrus*	?*P. polygyrus*	Con-San	Normandy	France (northern)	42
?*P. polygyrus*	?*P. polygyrus*	Con-San	Hauts-de-France	France (northern)	42
?*P. polygyrus*	?*P. polygyrus*	Cen	Hauts-de-France	France (northern)	31
P. rugosus	*P. rugosus*	Con	Hauts-de-France	France (northern)	40, 30
?*P. rugosus*	?*P. rugosus*	Con-San	Normandy	France (northern)	25, 30, 42
?*P. rugosus*	?*P. rugosus*	Con-San	Hauts-de-France	France (northern)	25, 30, 42
?*P. rugosus*	?*P. rugosus*	Con-San	Centre-Val de Loire	France (central)	42
?*P. rugosus*	?*P. rugosus*	Tur	Hauts-de-France	France (northern)	30
?*P. rugosus*	?*P. rugosus*	?Con-San	New Aquitaine	France (western)	43
P. mammillaris	*P. altior*	Cen-Con	Saxony	Germany (eastern)	17
P. decurrens	*P. decurrens*	Cen-Con	Saxony	Germany (eastern)	17
P. latissimus	*P. latissimus*	Cen-Con	Saxony	Germany (eastern)	17
P. paucisulcatus	*P. latissimus*	Con	Saxony	Germany (eastern)	25
P. decurrens	*P. decurrens*	Cen	Westphalia	Germany (western)	15
P. latissimus	*P. latissimus*	Tur	Westphalia	Germany (western)	8, 15, 48
?*P. latissimus*	?*P. latissimus*	Cen-Con	Westphalia	Germany (western)	7, 35
P. mammillaris	*P. mammillaris*	Tur	Westphalia	Germany (western)	15
P. latissimus	*P. cf. P. mediterraneus*	Tur	Westphalia	Germany (western)	15
P. polygyrus	*P. polygyrus*	?Cen-Tur	Westphalia	Germany (western)	15
?*P. belluccii*	?*P. polygyrus*	Cam	Westphalia	Germany (western)	35

Information Classification: General

TABLE S3. (Continued)

P. decurrens	*P. decurrens*	Cen	Veneto region	Italy (northeastern)	2, 6
P. decurrens	*P. decurrens*	Cen	Marche region	Italy (central)	10
P. altior	*P. altior*	Tur	Veneto region	Italy (northeastern)	3
P. rugosus	*P. rugosus*	San	Veneto region	Italy (northeastern)	3
P. latissimus	*P. latissimus*	Tur-?Cam	Veneto region	Italy (northeastern)	2, 5
P. decurrens	*P. decurrens*	?Cen-Tur	Sicily region	Italy (southern)	45
P. mediterraneus	*P. mediterraneus*	Tur	Veneto region	Italy (northeastern)	2, 4
P. polygyrus	*P. marginalis*	UCr	Veneto region	Italy (northeastern)	12
P. mammillaris	*P. mammillaris*	UCr	Veneto region	Italy (northeastern)	12
P. mortoni	*P. mortoni*	UCr	Veneto region	Italy (northeastern)	12
P. polygyrus	*P. polygyrus*	UCr	Veneto region	Italy (northeastern)	12
?*P. mammillaris*	?*P. mammillaris*	?Cen-Tur	Kaunas	Lithuania (central)	1, 13
?*P. elevatus*	?*P. altior*	?Tur-Con (37)	Skirsnemunė	Lithuania (central)	25
?*P. rugosus*	?*P. rugosus*	San-Cam	Kaunas	Lithuania (central)	13
P. latissimus	*P. latissimus*	UCr	Kaunas	Lithuania (central)	13
?*P. decurrens*	?*P. decurrens*	Cen	Kaunas	Lithuania (central)	13
P. mammillaris	*P.* cf. *P. altior*	UCr	Opole	Poland (southern)	46
P. cf. *rugosus*	*P.* cf. *P. altior*	Tur	Opole	Poland (southern)	44
P. mammillaris	*P. anonymus*	Tur	Opole	Poland (southern)	27, 36
P. decurrens	*P. decurrens*	Cen	Glanow	Poland (southern)	44
P. latissimus	*P. latissimus*	Tur	Opole	Poland (southern)	27, 36
P. polygyrus	*P. latissimus*	?Tur	Opole	Poland (southern)	27, 36
P. mammillaris	*P. mammillaris*	Cen	Sobkow	Poland (southern)	44
P. mammillaris	*P. mammillaris*	Tur	Opole	Poland (southern)	27, 36
?*P. mammillaris*	?*P. mammillaris*	Cen-?Tur	Bladen (now Włodzienin)	Poland (southern)	29

Information Classification: General

TABLE S3. (Continued)

P. polygyrus	*P. polygyrus*	Tur	Mydlniki	Poland (southern)	44
?*P. polygyrus*	?*P. polygyrus*	?Tur	Opole	Poland (southern)	29
?*P. polygyrus*	?*P. polygyrus*	Tur	Cracow	Poland (southern)	44
?*P. rugosus*	?*P. rugosus*	UCr	Cracow	Poland (southern)	44
P. rugosus	*P. altior*	Tur-Con	Ormeniş	Romania (central)	52
?*P. mammillaris*	?*P. mammillaris*	Tur-Con	Peştera	Romania (southeastern)	19
P. aff. *mammillaris*	*P. anonymus*	UCr	Ferriol-Elche	Spain (southeastern)	34
P. aff. *mammillaris*	*P. decurrens*	UCr	Ferriol-Elche	Spain (southeastern)	34
Ptychodus sp.	*P.* cf. *latissimus*	UCr	Ferriol-Elche	Spain (southeastern)	34
?*Ptychodus* sp.	?*Ptychodus* sp.	Cen	western Pyrenees	Spain (northern)	33
P. decurrens	*P. decurrens*	UCr	Annetorp, Oretorp	Sweden (southern)	14
P. mammillaris	*P. rugosus*	UCr	Annetorp, Oretorp	Sweden (southern)	14
P. mammillaris	*P. altior*	Cen-San	Oberriet	Switzerland (northeastern)	28
P. marginalis	*P. marginalis*	Cen-San	Oberriet	Switzerland (northeastern)	28
P. cf. *P. polygyrus*	*P.* cf. *P. polygyrus*	Cen-Tur	St. Gallen	Switzerland (northeastern)	24

Information Classification: General

TABLE S4. Literature Cited (**N**, reference numbers reported in Table S3 for the occurrences of *Ptychodus* from Europe).

N	References
1	Adnet, S., H. Cappetta, and R. Mertiniene. 2008. Re-evaluation of squaloid shark records from the Albian and Cenomanian of Lithuania. Cretaceous Research 29(4):711–722.
2	Amadori, M., J. Amalfitano, L. Giusberti, E. Fornaciari, and G. Carnevale. 2019a. Resti inediti di *Ptychodus* Agassiz, 1834 (Ptychodontidae, Chondrichthyes) conservati presso il Museo Civico di Rovereto (Trento). Annali del Museo Civico di Rovereto 34:221–247.
3	Amadori, M., J. Amalfitano, L. Giusberti, E. Fornaciari, V. Luciani, G. Carnevale, and J. Kriwet. 2019b. First associated tooth set of a high-cusped *Ptychodus* (Chondrichthyes, Elasmobranchii) from the Upper Cretaceous of northeastern Italy, and resurrection of *Ptychodus altior* Agassiz, 1835, Cretaceous Research 93:330–345.
4	Amadori, M., J. Amalfitano, L. Giusberti, E. Fornaciari, G. Carnevale, and J. Kriwet. 2020a. A revision of the Upper Cretaceous shark *Ptychodus mediterraneus* Canavari, 1916 from northeastern Italy, with a reassessment of *P. latissimus* and *P. polygyrus* Agassiz, 1835 (Chondrichthyes; Elasmobranchii). Cretaceous Research 110:104386.
5	Amadori, M., J. Amalfitano, L. Giusberti, E. Fornaciari, G. Carnevale, and J. Kriwet. 2020b. The Italian record of the Cretaceous shark, *Ptychodus latissimus* Agassiz, 1835 (Chondrichthyes; Elasmobranchii). PeerJ 8:e10167.
6	Amalfitano, J., L. Giusberti, E. Fornaciari, and G. Carnevale. 2020. Upper Cenomanian fishes from the Bonarelli level (OAE2) of Northeastern Italy. Rivista Italiana di Paleontologia e Stratigrafia 126(2):261–314.
7	Arnold, H. 1964. Fossilliste für die Münsterländer Oberkreide. Fortschritte in der Geologie von Rheinland und Westfalen 7:309–330.

Information Classification: General

TABLE S4. (Continued)

8	Brignon, A., 2019. Le *Diodon* devenu requin. L'histoire des premières découvertes du genre *Ptychodus* (Chondrichthyes). Bourg-la-Reine, 100 pp.
9	Bristow, R., R. Mortimore, and C. Wood. 1998. Lithostratigraphy for mapping the Chalk of southern England. Proceedings of the Geologists' Association 108(4):293–315.
10	Capasso, L. 2018. Implicazioni della presenza di *Ptychodus decurrens* Agassiz 1839 (Elasmobranchi, †Ptychodontidae) nel cretaceo Sup del Passo del Furlo, Italia Centrale. Thalassia Salentina 40:41–48.
11	Christensen, W. K. 1997. Paleobiogeography and migration in the Late Cretaceous belemnite family Belemnitellidae. Acta palaeontologica polonica 42(4):457–495.
12	D'Erasmo, G. 1922. Catalogo dei Pesci fossili delle Tre Venezie: Padova. Regia Università di Padova, Memoria dell'Instituto Geologico 6:1–181.
13	Dalinkevičius, J. A. 1935. On the Fossil Fishes of the Lithuanian Chalk: I. Selachii. Vytauto Didžiojo Universitetas Matematikos e Gamtos Fakulteto 9:245–305.
14	Davis, J. W. 1890. On the fossil Fish of the Cretaceous Formations of Scandinavia. The Royal Dublin Society 4(2), Williams and Norgate, Dublin, 434 pp.
15	Diedrich, C. G. 2013. Facies related phylostratigraphy of the benthic neoselachian *Ptychodus* from the Late Cretaceous (Cenomanian/Turonian) of the Pre-North Sea Basin of Europe. Cretaceous Research 41:17–30.
16	Dixon, F. 1850. The Geology and Fossils the Tertiary and Cretaceous Formations of Sussex. Longman, Brown, Green, and Longmans. Printed by Richard And John Edward Taylor, Red Lion Corrt, Fleet Street, London, [I–XVI] + 422 pp.
17	Fischer, J., I. Kogan, E. Popov, N. Janetschke, and M. Licht. 2017. The Late Cretaceous chondrichthyan fauna of the Elbtal Group (Saxony, Germany). Research and Knowledge 3(2):13–17.

Information Classification: General

TABLE S4. (Continued)

18	Friedman, M., H. T. Beckett, R. A. Close, and Z. Johanson. 2016. The English chalk and London clay: two remarkable British bony fish Lagerstätten. Geological Society, London, Special Publications 430(1):165–200.
19	Gallemí, J., I. Lazar, and G. López. 2011. Albian to Coniacian macrofaunal distribution around Ostrov and Medgidia (Southern Dobrogea, SE Romania): preliminary results. 8th Romanian Symposium on Paleontology:50-51.
20	Gervais, P. 1852. Zoologie et paléontologie françaises, 1st ed., tome 2. Arthus Bertrand, Libraire-Éditeur, Paris, 271 pp.
21	Goñi, I., and G. Cuny. 2022. New record of the genus *Ptychodus* Agassiz, 1834, (Chondrichthyes, Elasmobranchii) from the Upper Cretaceous of Bornholm (Denmark). Bulletin of the Geological Society of Denmark 70:19–25.
22	Graversen, O. 2004. Upper Triassic–Cretaceous stratigraphy and structural inversion offshore SW Bornholm, Tornquist Zone, Denmark. Bulletin of the Geological Society of Denmark 51:111–136.
23	Guinot, G., C. J. Underwood, H. Cappetta, and D. Ward. 2013. Sharks (Elasmobranchii: Euselachii) from the late Cretaceous of France and the UK. Journal of Systematic Palaeontology 11(6):589–671.
24	Hantke, R. 1958. *Ptychodus*-Zähne aus dem Seewerkalk von Grabs (Kt. St. Gallen). Geologisches Institut der Eidg. Technischen Hochschule und der Universität Zürich. Eclogae Geologicae Helvetiae 51:61-65.
25	Herman, J. 1977. Les Sèlaciens des terrains nèocrètacès et palèocènes de Belgique et des contrèes limitrophes. Elèments d'une biostratigraphie intercontinentale. Mèmoires pour servir à l'explication des Cartes gèologiques et minières de la Belgique 15:1–450.

Information Classification: General

TABLE S4. (Continued)

26	Hoyez, B., Girard, J. and N. Cottard. 2020. Le Cénomanien du littoral normand entre la Valleuse d'Antifer et le Cap de La Hève. Carnets de Géologie, Brest, 225 pp.
27	Jagt-Yazykova, E. A., and J. W. Jagt. 2015. Stratigraphy and faunal content of Turonian strata in the Opole area, southwest Poland. Field Guide, 28.
28	Kürsteiner, P., and C. Klug. 2018. Fossilien im Alpstein: Kreide und Eozän der Nordostschweiz, Appenzeller Verlag, Switzerland, 360 pp.
29	Leonhard, R. 1898. Die Fauna der Kreideformation in Oberschlesien. Palaeontographica 44:11–70.
30	Leriche, M. 1902. Révision de la faune ichthyologique des terrains crétacés du Nord de la France. Annales de la Societe Geologique du Nord 31:87–154.
31	Leriche, M. 1906. Contribution à l'étude des poissons fossiles du Nord de la France et des régions voisines. Annales de la Societe Geologique du Nord 35:338–357.
32	Longbottom, A. E. and C. Patterson. 1987. Fishes. In Smith A. B. (ed.), Fossils of the Chalk (Field Guides to Fossils, nr. 2), The Palaeontological Association:238–265.
33	López-Horgue, M., and F. Poyato-Ariza. 2005. Cretaceous fish record from the Basque-Cantabrian Basin, N. Spain: environments and palaeobiogeography. Servicio de Publicaciones de la Universidad Autónoma de Madrid/UAM Ediciones, Madrid. Fourth International Meeting on Mesozoic fishes, Extended Abstracts:161–165.
34	Mendiola, C. 2004. Primera cita española del género *Ptychodus* Agassiz 1839 (Chondrichthyes, Euselachii). Revista de la Societat Paleontológica d'Elx 13:1–14.
35	Müller, A. 1989. Selachier (Pisces: Neoselachii) aus dem höheren Campanium (Oberkreide) Westfalens (Nordrhein-Westfalen, NW-Deutschland). Landschaftsverband Westfalen-Lippe, Münster, 161 pp.

Information Classification: General

TABLE S4. (Continued)

36	Niedźwiedzki, R., and M. Kalina. 2003. Late Cretaceous sharks in the Opole Silesia region (SW Poland). Geologia Sudetica 35(1–76):13–24.
37	Olszewska-Nejbert, D. 2007. Late Cretaceous (Turonian–Coniacian) irregular echinoids of western Kazakhstan (Mangyshlak) and southern Poland (Opole). Acta Geologica Polonica 57(1):1–87.
38	Passy, A. 1832. Description géologique du département de la Seine-Inférieure. Imprimerie de Nicétas Periaux, Rouen, [iv] + xvi + 371 pp.
39	Plaks, D. P., and A. K. Grigorevich. 2017. On the findings of the Mesozoic and Palaeogene ichthyofauna in the territory of Belarus. Sovremennye problemy geohimii, geologii i poiskov mestorozhdenij poleznyh iskopaemyh: materialy Mezhdunar. nauch. konf., posvyashchennoj 110-letiyu so dnya rozhdeniya akademika K. I. Lukasheva, Minsk: Pravo i ekonomika:61–63.
40	Priem, F. 1896a. Sur des dents de poissons du Crétacé supérieur de France. Bulletin de la Société Géologique de France 24(1):288–295.
41	Priem, F. 1896b. Sur les poissons de la craie phosphatée des environs de Péronne. Bulletin de la Société Géologique de France 24(3):9–23.
42	Priem, F. 1908. Étude des poissons fossiles du bassin parisien. Annales de Paléontologie, 144 pp.
43	Priem, F. 1911. Étude des poissons fossiles du Bassin Parisien (supplément). Annales de Paléontologie 6:1–44.
44	Radwański, A., and R. Marcinowski. 1996. Elasmobranch teeth from the mid-Cretaceous sequence of the Mangyshlak Mountains, Western Kazakhstan. Acta Geologica Polonica 46(1–2):165–169.
45	Rindone, A. 2008. L'ittiofauna cretacica della Sicilia Nord-Orientale. Nota preliminare. Atti del Museo Civico di Storia Naturale di Trieste 53:49–66.

Information Classification: General

TABLE S4. (Continued)

46	Roemer, F. 1870. Geologie von Oberschlesien, Atlas. Druck von R. Nischkowsky, Breslau, 101 pp.
47	Rowe, A. W. 1900. The zones of the White Chalk of the English coast. I. Kent and Sussex. Proceedings of the Geologists' Association 16:289–368.
48	Schlotheim, E. F. Von 1822. Nachträge zur Petrefactenkunde. Becker'schen Buchhandlung, Gotha, xii + 100 pp.
49	Schultz, O., and W. E. Piller. 2013. Catalogus Fossilium Austriae. Ein systematisches Verzeichnis aller auf österreichischem Gebiet festgestellten Fossilien. Band 3. Pisces, Verlag der Österreichischen Akademie der Wissenschaften, Wien, 578 + XXXVII pp.
50	Sternberg, K. M. von 1827. Řeč Präsidenta hraběte Kašpara Šternberka. Časopis Společnosti wlastenského museum w Čechách 1(3):23–36.
51	Trbušek, J. 1999. Upper Cretaceous sharks and rays from the Prokop opencast mine at Březina near Moravská Třebová. Acta Universitatis Palackianae Olomucensis, Facultas Rerum Naturalium, Geologica 36:51–61.
52	Trif, N., and V. A. Codrea. 2017. Ocurente Ale Genului Mezozoic *Ptychodus* (Elasmobranchii) În România: Date Preliminare. Al XV-lea Simpozion National Student esc "GEOECOLOGIA":7–11.
53	Vullo, R., and E. Arnaud. 2009. Présence de *Ptychodus latissimus* Agassiz, 1843 (Elasmobranchii, Hybodontiformes) dans le Crétacé Supérieur des Charentes. Société des sciences naturelles de la Charente-Maritime 9(9):962–966.
54	Wiese, F., S. Čech, B. Ekrt, M. Košt'ák, M. Mazuch, and S. Voigt. 2004. The Upper Turonian of the Bohemian Cretaceous Basin (Czech Republic) exemplified by the Úpohlavy working quarry: integrated stratigraphy and palaeoceanography of a gateway to the Tethys. Cretaceous Research 25(3):329–352.

Information Classification: General

TABLE S4. (Continued)

55	Woodward, A. S. 1912. The fossil fishes of the English Chalk, Part VII. Monograph of the Palaeontographical Society 65(320), London, i–viii + 225–264.
56	Woodward, J. 1729. An attempt towards the natural history of the fossils of England; in a catalogue of the English fossils in the collection of J. Woodward, M. D. Tome 1, part 2. F. Fayram, London, viii + 115 pp.

Information Classification: General
